# Awakening the natural capability of psicose production in *Escherichia coli*

**DOI:** 10.1038/s41538-023-00231-0

**Published:** 2023-10-14

**Authors:** Jayce E. Taylor, Dileep Sai Kumar Palur, Angela Zhang, Jake N. Gonzales, Augustine Arredondo, Timothy A. Coulther, Amiruddin Bin Johan Lechner, Elys P. Rodriguez, Oliver Fiehn, John Didzbalis, Justin B. Siegel, Shota Atsumi

**Affiliations:** 1 Department of Chemistry, University of California, Davis, Davis, CA 95616 USA; 2 Plant Biology Graduate Group, University of California, Davis, Davis, CA 95616 USA; 3 Genome Center, University of California, Davis, Davis, CA 95616 USA; 4 West Coast Metabolomics Center, UC Davis Genome Center, University of California, Davis, Davis, CA 95616 USA; 5 Mars, Incorporated, 6885 Elm Street, McLean, VA 22101 USA; 6 Department of Biochemistry and Molecular Medicine, University of California, Davis, Sacramento, CA 95616 USA

**Keywords:** Applied microbiology, Metabolic engineering

## Abstract

Due to the rampant rise in obesity and diabetes, consumers are desperately seeking for ways to reduce their sugar intake, but to date there are no options that are both accessible and without sacrifice of palatability. One of the most promising new ingredients in the food system as a non-nutritive sugar substitute with near perfect palatability is D-psicose. D-psicose is currently produced using an in vitro enzymatic isomerization of D-fructose, resulting in low yield and purity, and therefore requiring substantial downstream processing to obtain a high purity product. This has made adoption of D-psicose into products limited and results in significantly higher per unit costs, reducing accessibility to those most in need. Here, we found that *Escherichia coli* natively possesses a thermodynamically favorable pathway to produce D-psicose from D-glucose through a series of phosphorylation-epimerization-dephosphorylation steps. To increase carbon flux towards D-psicose production, we introduced a series of genetic modifications to pathway enzymes, central carbon metabolism, and competing metabolic pathways. In an attempt to maximize both cellular viability and D-psicose production, we implemented methods for the dynamic regulation of key genes including clustered regularly interspaced short palindromic repeats inhibition (CRISPRi) and stationary-phase promoters. The engineered strains achieved complete consumption of D-glucose and production of D-psicose, at a titer of 15.3 g L^-1^, productivity of 2 g L^-1^ h^-1^, and yield of 62% under test tube conditions. These results demonstrate the viability of whole-cell catalysis as a sustainable alternative to in vitro enzymatic synthesis for the accessible production of D-psicose.

## Introduction

The deadly rise in sedentary lifestyle and access to calorie-dense foods has tripled global obesity rates from 1975 to 2016^1^. Almost 40% of adults world-wide are considered overweight, a major risk factor associated with cardiovascular disease, diabetes, musculoskeletal disorders, and some cancers^1^. Within the United States alone, nearly 50% of adults are predicted to have obesity by 2030, with almost 25% of adults having severe obesity^2^. In the U.S., increases in severe obesity is predicted to disproportionally affect women, Black non-Hispanic adults, and adults with household income <$50,000, which could lead to further socioeconomic disparities as a result of obesity-associated medical complications, chronic disease, lower life expectancy, and weight stigma^2^. One critical factor in combating this issue is in the development of new tools and cost-effective ingredients that enable the food industry to play a role in providing nutritionally balanced products. One of the most immediate impacts that can be made is through the substitution of sucrose and high-fructose corn syrup with zero and low-calorie sugar substitutes. As such, the market for sugar substitutes is expected to reach 20.6 billion USD by 2025^3^. However, this cannot be done in a manner that sacrifices enjoyment or cost for the customer. One of the most promising approaches to thread this delicate needle appears to be through the use of rare sugars.

Rare sugars are monosaccharides uncommonly found in nature, which differ slightly in structure from common sugars such as glucose and fructose. Many rare sugars are non-nutritive and possess potential health benefits such as anti-inflammatory, anti-viral, and tumor-suppressing capabilities, making them intriguing targets for pharmaceuticals and as sugar substitutes in food items^4^. They are being studied as sustainable alternatives to herbicides, fungicides, insecticides, and plant growth and immune system modulators^4^. One such rare sugar, D-psicose, is a Generally Recognized as Safe, zero-calorie, C3 epimer of D-fructose, with 70% the sweetness of sucrose^5^. Marketed to consumers as “allulose”, D-psicose has a desirable flavor profile along with favorable browning, hygroscopic, and solubility properties. Additionally, studies have correlated consumption of D-psicose to antihyperglycemic, antihyperlipidemic, antiparasitic, and antioxidant health benefits^4^. As with most rare sugars, a limitation to the study of D-psicose and its implementation in industry is the lack of economic, large-scale production. D-Psicose is naturally found in fruits, wheat, and syrups, but only in minute concentrations^6^. D-Psicose, along with other rare sugars, could be extracted from their natural sources, but their low abundance would lead to costly purification and the waste of consumable biomass.

**Fig. 1:**
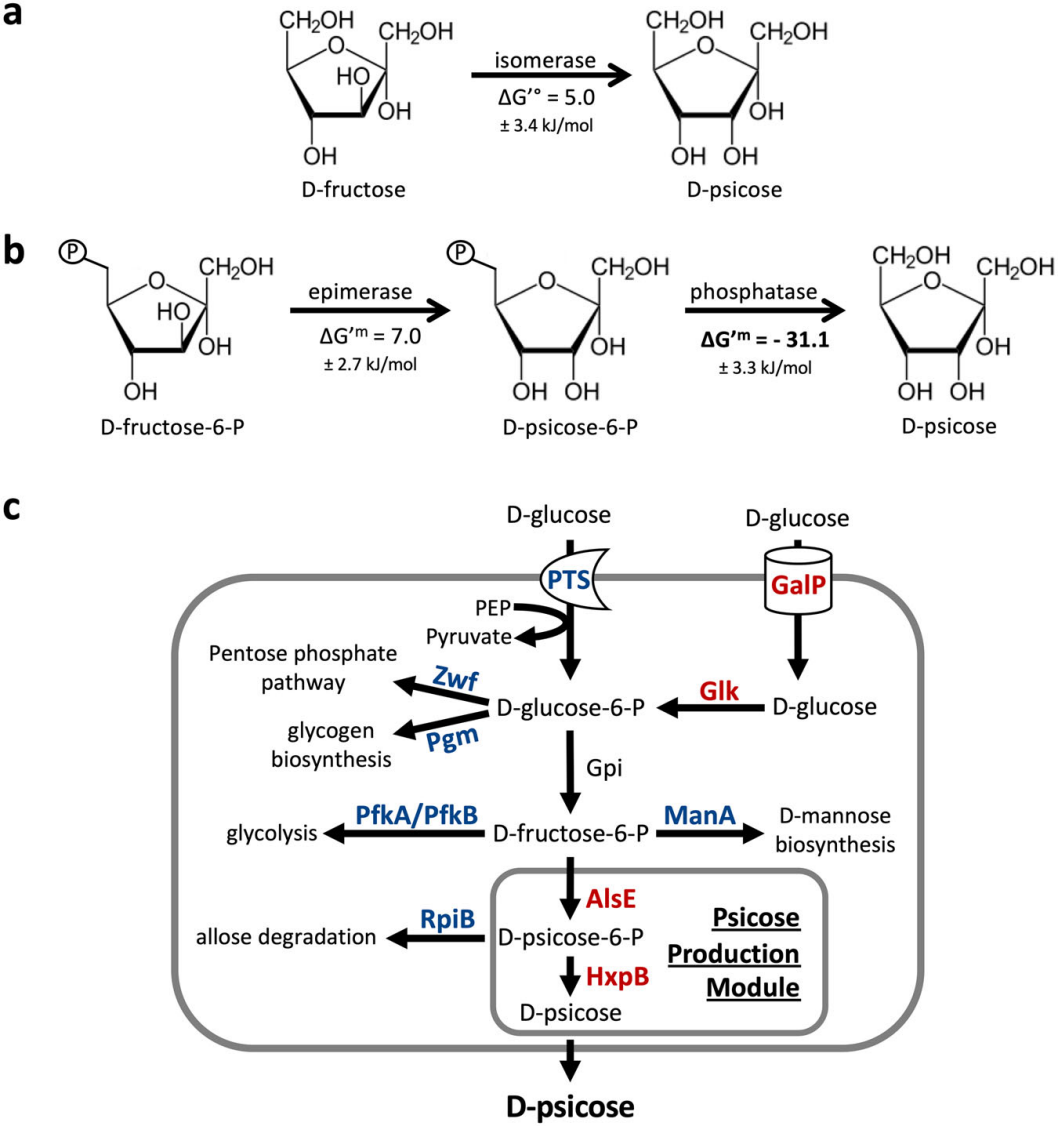
Strategies for the biosynthesis of D-psicose. **a** The current industrial method for D-psicose production leads to limited yield (~50%) due to a positive ΔG’°. **b** In the proposed biosynthetic pathway of D-psicose, the dephosphorylation step thermodynamically drives production forwards due to a large negative ΔG’^m^ under cellular reactant concentrations of 1 mM. **c** The proposed pathway for the biosynthetic production of D-psicose in *E. coli*. Deleted steps are in blue. Overexpressed steps are in red. PTS, the phosphotransferase system; AlsE, D-allulose 6-phosphate 3-epimerase, HxpB hexitol phosphatase B.

Current commercial production processes of D-psicose utilize a combination of D-tagatose-3-epimerase (DTEase) and D-psicose-3-epimerase (DPEase) to catalyze the conversion from fructose^7–9^. Both enzymes operate under high temperature and alkaline pH to epimerize the C3 carbon of D-fructose to form D-psicose^8^. Attempts to bolster production have focused on engineering these enzymes for improved catalytic efficiency, thermostability, and ability to perform at lower pHs and temperatures^4,10^. Despite concerted efforts to improve DPEases and DTEases, this method inherently suffers from limited yield due to a lack of thermodynamic driving force, and therefore has not achieved conversion yields greater than 50%^8,10^. The epimerization of D-fructose to D-psicose is reversible and has a predicted ΔG’° of +5 kJ mol^-1^, making it thermodynamically unfavorable and more likely to favor D-fructose at equilibrium (Fig. 1a)^11^. Low conversion results in a mixed solution of D-fructose and D-psicose, making isolation and purification by simulated moving bed chromatography or anion exchange resin a major challenge^10,12^. Methods that achieved conversions greater than 50% relied on the inclusion of toxic or expensive cofactors^10^.

To overcome this barrier, phosphorylation and dephosphorylation can be used to provide thermodynamic incentive and a driving force towards D-psicose production (Fig. 1b). Carrying a predicted ΔG^’m^ of −31.1 kJ mol^-1^ at typical physiological concentrations of 1 mM, the dephosphorylation of D-psicose-6-phosphate (P6P) to D-psicose is a highly favorable reaction^11^. Dephosphorylation has been utilized as a thermodynamic sink for an in vitro enzyme system to produce D-psicose from the polysaccharide maltodextrin^13^. This system required five core and four auxiliary enzymes operating at elevated temperatures and resulted in a mixed solution of D-glucose, D-fructose, and D-psicose. Although successful at generating some D-psicose, this system suffered from the same limitations as other in vitro systems including enzymes which only operate at high temperatures, the need to isolate and purify pathway enzymes, and reliance on phosphorylating agents or polysaccharides to provide energy for phosphorylation. Additionally, D-fructose was generated as a side product and unable to be reassimilated back into the production pathway, which would lead to increased separation and purification cost in an industrial setting. As such, phosphorylation/dephosphorylation is more suited for whole-cell catalysis, where the enzymatic machinery and cofactors are readily available and side products such as D-fructose can be reused by the cells. In whole-cell catalysis, living cells play the role of chemical factory by assembling enzymes for chemical production, providing cofactors for enzymes to operate, and excreting products for simplified purification, all under environmentally friendly production conditions and temperatures^14–17^. Feedstocks for living cells can consist of sugars from agricultural waste, reducing competition with commercial food production. The technology and infastructure required for whole-cell catalysis and fermentation is established on an industrial scale, including for the model organism, *Escherichia coli*. Like most organisms, *E. coli* utilizes phosphorylation/dephophorylation of sugars as part of sugar consumption and central carbon metabolism^18^. In an attempt to overcome the inherent limitations of in vitro biosynthesis, a biosynthetic pathway using heterologous enzymes was constructed in *E. coli* to produce D-psicose from D-fructose^19^. However, cultures produced a minimal amount of D-psicose (~1.6 g L^-1^) and suffered from poor growth, even when supplemented with glycerol as an additional carbon source. Most recently, the supplementation of ATP and sodium hexametaphosphate was shown to improve D-psicose production from D-glucose in *E. coli*^20^. However, the supplementation of ATP and sodium hexametaphosphate drastically increases production cost, and the system did not consume all glucose and produced D-fructose as a side product, which would increase the cost of downstream processing. As demonstrated by the studies listed above, in vivo D-psicose production has great potential, but further research into enzyme selection, carbon flux regulation, feedstock optimization, and balancing energetics are required to achieve industrially relevant levels of production. Through our research, we attempt to address all four of these subjects.

In this study, we discovered that *E. coli* is capable of producing D-psicose from D-glucose without the expression of exogenous genes (Fig. 1b). Within *E. coli*, carbon metabolism begins with the phosphotransferase system (PTS), where D-glucose is simultaneously phosphorylated and transferred across the cell membrane^18^. Alternatively, glucose can be transported across the cell membrane by galactose proton symporter GalP, after which it is phosphorylated by glucokinase Glk^21,22^. D-glucose-6-phosphate (G6P) is then isomerized to D-fructose-6-phosphate (F6P), which while typically directed into glycolysis, we diverted towards D-psicose production using static and dynamic carbon flux regulation techniques^18^. F6P can be epimerized to P6P using the native D-allulose-6-phosphate 3-epimerase (AlsE), which is then dephosphorylated to D-psicose using the phosphatase hexitol-phosphatase B (HxpB) and excreted from the cell. By utilizing *E. coli’s* native genes, we successfully generated D-psicose from D-glucose at yields greater than 50%, without the need for heterologous enzyme expression.

We further improved the capacity of *E. coli* to produce D-psicose by additionally expressing *alsE* and *hxpB* and removing or regulating competing metabolic pathways, including the pentose phosphate pathway (PPP), glycogen biosynthesis, glycolysis, the D-allose degradation pathway, and the D-mannose degradation pathway. D-glucose import was supplemented by additionally expressing the native galactose proton symporter gene, *galP*, and the glucokinase gene, *glk*. To further increase production while maintaining cellular viability, we explored multiple strategies for dynamic gene regulation and carbon flux partitioning. During the growth phase, cells need more energy to rigorously grow, and genes related to glycolysis should be expressed. When the cells enter the stationary phase and are not actively growing, carbon flux can be diverted from glycolysis to D-psicose production. To balance carbon partitioning depending on growth phase, we explored two strategies to dynamically regulate the expression of key metabolic and D-psicose production genes: inducer-free stationary-phase promoters^23–25^ and clustered regularly interspaced short palindromic repeats interference (CRISPRi)^26,27^.

## Results and discussion

### Design of a thermodynamically favorable D-psicose production pathway

Currently, the primary method for production of D-psicose involves the in vitro enzymatic isomerization of D-fructose to D-psicose, a thermodynamically unfavorable process that results in incomplete product formation (~50%) and requires expensive purification (Fig. 1a)^10^. To overcome the thermodynamic limitations of this method, we proposed a pathway driven by the phosphorylation and dephosphorylation of sugars, a process whose required cofactors and enzymes are readily available within living organisms (Fig. 1b, c). Therefore, we chose the model organism *E. coli* to host the D-psicose biosynthetic pathway.

The proposed pathway begins with the assimilation of D-glucose into *E. coli* via the PTS^18^, which converts D-glucose to G6P (Fig. 1c). Alternatively, D-glucose can be assimilated by the galactose proton symporter GalP^21^, after which it is phosphorylated to G6P by glucokinase Glk^22^ (Fig. 1c). G6P is then isomerized to F6P via glucose 6-phosphate isomerase (Gpi)^18^. Here, the proposed pathway diverges from native carbon metabolism. Many enzymes are promiscuous, utilizing a variety of substrates. As such, we theorized *E. coli* may natively possess enzymes capable of producing D-psicose given the right conditions and alterations to sugar metabolism. F6P could be epimerized to P6P, which could then be dephosphorylated in a final, thermodynamically favorable step to D-psicose (Fig. 1c). We theorized that placing this favorable reaction at the end of our biosynthetic pathway drives flux through the production pathway by way of restoring equilibrium^28,29^. As P6P is favorably dephosphorylated to D-psicose and excreted from the cell, equilibrium is restored by generating more P6P from F6P.

### Assessing *E. coli’s* native D-psicose production capabilities

**Fig. 2:**
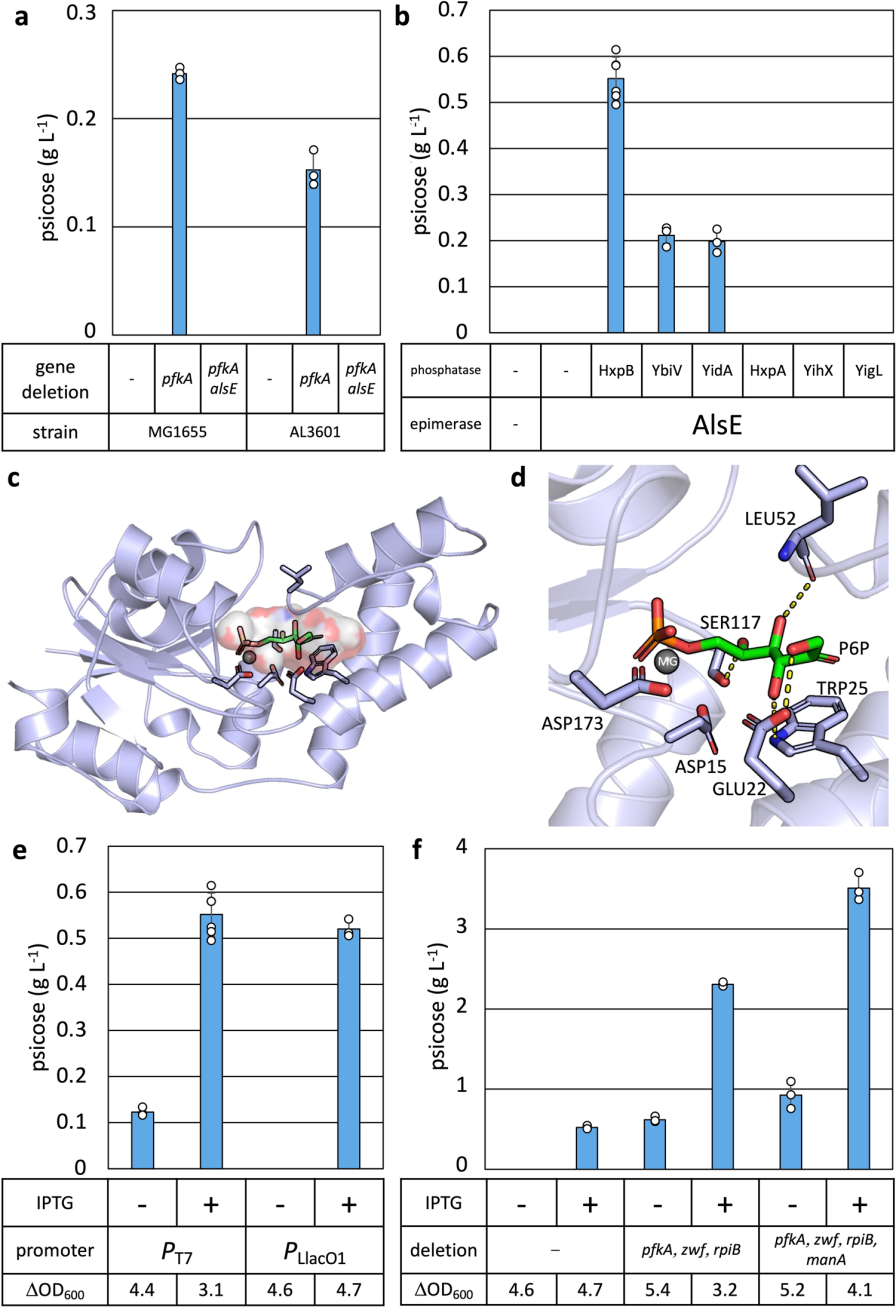
D-psicose production capability in *E. coli*. Cells were grown in M9P media with 10 g L^-1^ glucose at 37 °C to OD_600_ ~ 0.4, then grown at 30 ˚C for 24 h. At OD_600_ ~ 0.4, 1 mM IPTG was added (**a, b, e, f**). **a** D-psicose production in MG1655 and AL3601 (Supplementary Table 1) with and without the deletion of *pfkA* and/or *alsE*. **b** Various sugar phosphatases with AlsE were tested for D-psicose in AL3601. **c** The AlphaFold predicted structure of HxpB with P6P (199 Å^3^) located in the active site pocket (429 Å^3^), with volumes calculated using MoloVol and CAVER, respectively^66,67^. **d** ASP173 interacts with the magnesium ion, which in turn positions the phosphate in P6P for nucleophilic attack by ASP15. Residues GLU22, TRP25, LEU52, SER117 are predicted to form hydrogen bonds (shown as dotted yellow lines) with the hydroxyl groups and position the P6P for hydrolysis. **e** The operon of *alsE* and *hxpB* was expressed under *P*_T7_ and *P*_LlacO1_ in AL3601 and AL1050 (Supplementary Table 1) respectively. ΔOD_600_ indicates the difference in OD_600_ at 0 h and 24 h. **f** Comparison of the effect of gene deletions on the production of D-psicose. Error bars indicate s.d. (*n* = 3 biological replicates).

To assess *E. coli*’s native capability of producing D-psicose, we tested production in *E. coli* MG1655 and MG1655-derived strain AL3601, which carries the gene encoding for T7 RNA polymerase^30^, in M9P media (Supplementary Table 1, *Methods*). Without any genetic manipulations, neither strain produced detectable levels of D-psicose (Fig. 2a).

We hypothesized that carbon flux must be purposely directed towards D-psicose production by accumulating the upstream metabolite, F6P (Fig. 1c). The major metabolic pathway competing for F6P is glycolysis, as F6P is preferentially converted to D-fructose 1,6-bisphosphate by phosphofructokinase A and B (PfkA and PfkB, Fig. 1c). To build up F6P pools within the cell, the gene encoding PfkA, which accounts for about 90% of phosphofructokinase activity^31,32^, was deleted in MG1655 and AL3601, generating AL4058 and AL3694 respectively (Supplementary Table 1). ∆*pfkA* strains produced D-psicose at 0.24 g L^−^^1^ in AL4058 and 0.15 g L^−1^ in AL3694, indicating that *E. coli* contains the enzymes necessary for producing D-psicose, most likely from F6P (Fig. 2a). D-psicose production was not detected in cultures grown in M9P media without glucose.

### Elucidating enzymes involved in D-psicose production

We identified D-allulose 6-phosphate 3-epimerase (AlsE) as a potential candidate for the conversion of F6P to P6P^33^. AlsE assimilates D-psicose into central carbon metabolism by converting P6P to F6P. Under high concentrations of F6P, AlsE has shown reverse activity and is capable of converting F6P to P6P^33^. To confirm whether AlsE is involved in the production of D-psicose, *alsE* was deleted in AL3694 and AL4058, resulting in strains AL4063 and AL4082 (Supplementary Table 1). Neither strain produced detectible levels of D-psicose, suggesting that AlsE is the epimerase involved in the production of D-psicose (Fig. 2a).

*E. coli* has a number of phosphatase enzymes with potential activity towards P6P^34^. We chose candidate phosphatases with a wide range of activity towards various sugar substrates and tested hexitol phosphatase B (HxpB), sugar phosphatase YbiV, sugar phosphatase YidA, hexitol phosphatase A (HxpA), α-D-glucose-1-phosphate phosphatase YihX, and phosphosugar phosphatase YigL^34^. To test each phosphatase for the conversion of P6P to D-psicose, genes for each phosphatase were individually expressed from *P*_T7_^35^, along with *alsE* on an expression plasmid (Supplementary Table 2). The plasmids were introduced to AL3601 (*pfkA*^+^). After 24 h, the strain harboring pAL1946 (*P*_T7_:*alsE-hxpB*) produced the most D-psicose, at 0.55 g L^−^^1^, suggesting that HxpB is an excellent phosphatase candidate for D-psicose production (Fig. 2b). The strain harboring pAL1947 (*P*_T7_:*alsE-ybiV*) or pAL2351 (*P*_T7_:*alsE-yidA*) produced 0.21 g L^−1^ and 0.20 g L^−1^ of D-psicose respectively, while cultures containing pAL2348 (*P*_T7_:*alsE-hxpA*), pAL2352 (*P*_T7_:*alsE-yihX*), and pAL2349 (*P*_T7_:*alsE-yigL*) did not generate detectable D-psicose (Fig. 2b).

### Identification of critical P6P binding motifs

A combination of AlphaFold^36^ and the Rosetta Molecular Suite^37^ were utilized to evaluate predicted binding modes between each of the six phosphatases and P6P. Phosphatases with activity towards P6P (HxpB, YbiV, and YidA, Fig. 2b) were predicted to form at least two internal hydrogen bonds and one additional hydrogen bond with the terminal hydroxyl group of P6P (Fig. 2c, d and Supplementary Fig. 1). Conversely, phosphatases without activity towards P6P (HxpA, YihX, and YigL, Fig. 2b), were predicted to not form hydrogen bonds with the terminal hydroxyl group of P6P (Supplementary Fig. 1). Phosphatase HxpB, which produced the highest D-psicose titer (Fig. 2b), was predicted to form hydrogen bonds between active site residues and the four hydroxyl groups of P6P (Fig. 2d). These predictions show that a minimum of three hydrogen bonds, including a hydrogen bond to the terminal hydroxyl group, is essential for binding P6P in a catalytically competent orientation. An example of the input files used, including constraints, RosettaScripts XML, and ligand params can be found in the Supplementary Information.

### Comparing expression system for D-psicose production

In AL3601, the T7 RNA polymerase expression system, which includes the T7 RNA polymerase (RNAP) gene under an IPTG-inducible *P*_lacUV5_ promoter^30^, was utilized. However, we observed decreased growth when IPTG was added to induce expression of T7 RNAP (Fig. 2e). Thus, an alternative expression system in which the operon of *alsE* and *hxpB* was expressed from the IPTG-inducible promoter *P*_LlacO1_ was tested^38^. Under induced conditions (with IPTG), the strain with *P*_LlacO1_:*alsE-hxpB* produced D-psicose similarly to the strain with *P*_T7_:*alsE*-*hxpB* (Fig. 2e). Importantly, the strain with *P*_LlacO1_:*alsE-hxpB* showed better repression under uninduced conditions (without IPTG) and did not have a growth burden under induced conditions compared to the strain with *P*_T7_:*alsE*-*hxpB* (Fig. 2e). Thus, the *P*_LlacO1_ expression system was used for further studies.

### Increasing D-psicose production by removing competing pathways

*E. coli* relies on two primary glycolytic pathways to metabolize glucose: the PPP and glycolysis (also known as the Embden Meyerhof Parnas pathway)^31^. The branching point between D-psicose production and the PPP occurs when glucose-6-phosphate dehydrogenase (Zwf) converts G6P to 6-phospho-D-glucono 1,5-lactone (Fig. 1c)^39^. The branching point between D-psicose production and glycolysis occurs when one of two phosphofructokinases, PfkA or PfkB, converts F6P to D-fructose 1,6-bisphosphate (Fig. 1c)^31^. We established that the deletion of *pfkA* leads to the development of D-psicose production in comparison to our unmodified base strains (Fig. 2a).

In addition to the PPP and glycolysis, the allose degradation pathway has potential to divert carbon flux away from D-psicose production by reassimilating P6P back into central carbon metabolism^40^. The *rpiB* gene encodes for allose-6-phosphate isomerase (RpiB), which may be able to convert P6P to aldehydo-D-allose 6-phosphate (Fig. 1c).

To redirect carbon flux away from central carbon metabolism and towards D-psicose production, gene knockouts ∆*pfkA*, Δ*zwf*, and Δ*rpiB* were constructed in AL1050, resulting in Strain 1 (Table 1). Those three deletions resulted in a fourfold increase in D-psicose production at 2.31 g L^−1^ of D-psicose (Fig. 2f).

**Table 1:** List of key strains used in this study.

Strain no.	*E. coli* strain	Key genotype	Plasmid	Plasmid contents
1	AL3756	Δ*pfkA* Δ*zwf* Δ*rpiB*	pAL2001	*P*_LlacO1_:*alsE-hxpB*
2	AL3990	1 + Δ*manA*	pAL2001	*P*_LlacO1_:*alsE-hxpB*
3	AL3990	1 + Δ*manA*	pAL2247	*P*_*gadB*_:*alsE-hxpB*
4	AL3990	1 + Δ*manA*	pAL2247 pAL2264	*P*_*gadB*_:*alsE-hxpB, P*_LlacO1_:*galP-glk*
5	AL4186	2 + Δ*pgm*	pAL2247 pAL2264	*P*_*gadB*_:*alsE-hxpB, P*_LlacO1_:*galP-glk*
6	AL4186	2 + Δ*pgm*	pAL2247 pAL2264 pAL2182	*P*_*gadB*_:*alsE-hxpB, P*_LlacO1_:*galP-glk, P*_tet_:*dcas9* pTargetF*-no target*
7	AL4186	2 + Δ*pgm*	pAL2247 pAL2264 pAL2188	*P*_*gadB*_:*alsE-hxpB, P*_LlacO1_:*galP-glk, P*_tet_:*dcas9* pTargetF*-pfkB*

All strains and plasmids used in this study are listed in Supplementary Tables 1 and 2, respectively.

Through knocking out *pfkA*, *zwf*, and *rpiB*, carbon flux was channeled into the D-psicose production pathway. In particular, knocking out *pfkA* and *zwf* should lead to an increase in F6P availability within cells^31^. These results reaffirmed our suspicions that F6P plays a key role as precursor to P6P, and that accumulation of F6P is necessary to drive carbon flux through the production pathway.

### Identification of a side product

When analyzing samples from Strain 1, we observed a significant peak on the high-performance liquid chromatography (HPLC) chromatogram that did not align with D-glucose, D-fructose, or D-psicose standards, or any media component. Using gas chromatography–mass spectrometry (GC-MS) analysis, we found the retention time and mass spectrum of the unknown peak matched the retention time and spectrum of D-mannose (Supplementary Fig. 2).

Under standard conditions, the D-mannose pathway involves the assimilation of D-mannose 6-phosphate (M6P) into central carbon metabolism by the reversible isomerization of M6P to F6P via mannose-6-phosphate isomerase (ManA)^41^. An accumulation of F6P could cause this reaction to run in reverse, generating M6P and D-mannose as a result. To test the hypothesis that this side product was D-mannose, we deleted *manA* in Strain 1, resulting in Strain 2 (Table 1).

The deletion of *manA* resulted in a considerable decrease in D-mannose production. Strain 2 with IPTG generated only 0.69 g L^−^^1^ of D-mannose compared to Strain 1 with IPTG, which produced 2.49 g L^−1^ of D-mannose (Supplementary Fig. 3). Complementary to the reduction in D-mannose production, the production of D-psicose in Strain 2 was 1.5 times higher than in Strain 1 (Fig. 2f). While not fully eliminated, the unwanted production of D-mannose was greatly decreased by the deletion of *manA*. Subsequent production was therefore carried out in strains containing Δ*pfkA*, Δ*zwf*, Δ*rpiB*, and Δ*manA*.

Although the D-mannose pathway was eliminated, there may be other D-fructose epimer pathways continuing to compete for carbon flux. Eliminating the PPP and limiting glycolysis can cause a substantial increase in cellular F6P pools, which if not funneled efficiently into D-psicose biosynthesis, may be acted upon by other epimerases or isomerases. In short, increased F6P availability may allow enzymes not normally observed to engage with the substrate and generate other sugar products.

### Utilization of a stationary phase promoter

Constructing production pathways within a microorganism requires careful carbon partitioning between essential metabolic processes and production, especially when working around central carbon metabolism^42–44^. Within our system, dynamically balancing carbon flux between glycolysis and the D-psicose pathway is critical for maximizing both cellular viability and D-psicose production.

The life cycle of an *E. coli* culture includes 5 distinct phases: lag, logarithmic, stationary, death, and long-term stationary phase^23^. The lag phase occurs when cells are inoculated into media and adjust their metabolic processes according to their new environment. Given favorable conditions, cells will rapidly grow and divide, entering the logarithmic phase. It is at this time that enzymes related to central carbon metabolism are most important, and the transcription of corresponding genes will be upregulated. Once the cells sense environmental stressors such as scarcity of media nutrients, their growth and division slows, and the culture enters the stationary phase. Here, culture density plateaus and genes related to stress response are expressed^24,45^. The transcription of these genes is regulated in part by the σ^38^-subunit of RNA polymerase, which recognizes the promoter region of a gene^23^.

We chose to utilize *E. coli*’s native gene regulatory system to balance carbon flux by affixing the D-psicose production genes, *alsE* and *hxpB*, downstream of a stationary phase-active promoter^23–25^. This should prevent the production pathway from competing with central carbon metabolism for carbon flux during the logarithmic phase of growth, a time when cells need carbon to rigorously grow and divide.

Four promoters previously shown to have activity during the stationary phase were chosen for testing using Green Fluorescent Protein as a reporter: *P*_*gadB*_, *P*_*cbpA2*_, *P*_*ihfA4*_, and *P*_*dps*_^23–25^. Each promoter was cloned upstream of *sfgfp* on an expression plasmid (Supplementary Table 2). The strongest promoter, *P*_*gadB*_, had an expression strength 100 times as strong as IPTG-induced promoter *P*_LlacO1_, in addition to having an expression time correlating to late logarithmic or early stationary phase (Supplementary Fig. 4a). The second strongest promoter, *P*_*cbpA2*_, closely followed the expression timing of induced *P*_LlacO1_, but with approximately two-thirds the expression strength (Supplementary Fig. 4b). *P*_*ihfA4*_ and *P*_*dps*_ also followed the expression timing of *P*_LlacO1_, but with lower expression strength than *P*_*cbpA2*_. (Supplementary Fig. 4c).

**Fig. 3:**
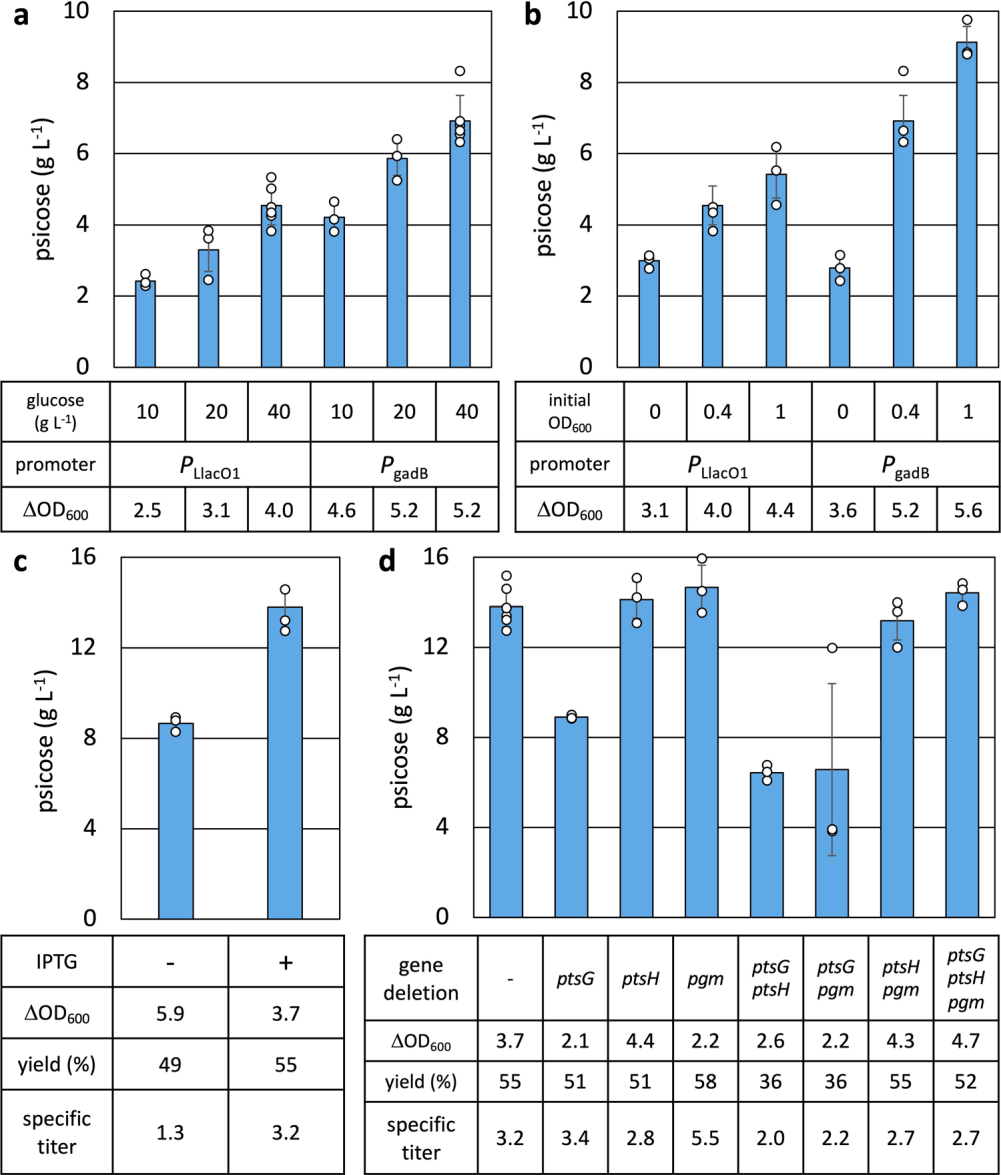
Enhancing D-psicose production capability in *E. coli*. **a** Cells were grown in M9P media with various concentrations of glucose to OD_600_ ~ 0.4 at 37 °C, then grown at 30 ˚C for 24 h. At OD_600_ ~ 0.4, 1 mM IPTG was added for the *P*_LlacO1_ constructs. The operon of *alsE* and *hxpB* was expressed under *P*_LlacO1_ (pAL2001) and *P*_gadB_ (pAL2247) in AL3756 and AL3990 respectively (Strain 1 and 2, Table 1). **b** Strain 1 and 2 were grown in M9P media with 40 g L^−1^ glucose at 37 °C to OD_600_ of ~0 (no culturing at 37 °C), ~0.4, or ~1, then grown at 30 ˚C for 24 h. When the temperature was shifted to 30 ˚C, 1 mM IPTG was added for the *P*_LlacO1_ constructs. **c** The operon of *galP* and *glk* were expressed under *P*_LlacO1_ (pAL2264, Supplementary Table 2). Strain 4 (AL3990 with pAL2264 and pAL2247, Table 1) was grown in M9P media with 40 g L^−^^1^ glucose to OD_600_ ~ 1, then grown at 30 ˚C for 24 h. At OD_600_ ~ 1, 1 mM IPTG was added to induce *P*_LlacO1_:*galP-glk*. Specific titer (g^−1^ L^−1^ OD_600_^−1^) indicates titer per final OD_600_. **d** Comparison of the effect of gene deletions on the production of D-psicose. *ptsG*, *ptsH*, and/or *pgm* were deleted in Strain 4. D-psicose production was done as described in (**c**). Error bars indicate s.d. (*n* = 3 biological replicates).

Due to its strong expression during the stationary phase, *P*_*gadB*_ was used to express the operon of *alsE* and *hxpB* (pAL2247, Supplementary Table 2). To evaluate the effects of the initial glucose concentration, we tested D-psicose production with 10, 20, and 40 g L^−^^1^. Under varying glucose concentrations (10, 20, and 40 g L^−1^) Strain 3 (*P*_*gadB*_:*alsE*-*hxpB*, Table 1) consistently produced greater titers of D-psicose and grew with a greater ΔOD_600_, which represents the difference in optical density at 600 nm (OD_600_) at 0 and 24 h, compared to Strain 2 (*P*_LlacO1_:*alsE*-*hxpB*, Fig. 3a). The highest D-psicose titers were achieved at a glucose concentration of 40 g L^−1^, which allowed Strain 3 to produce 6.92 g L^−1^ of D-psicose and grow with a ΔOD_600_ of 5.2, while Strain 2 produced 4.55 g L^−1^ and grew with a ΔOD_600_ of 4.0. The initial glucose concentration of 40 g L^−1^ was used for further studies.

Next, the impact of timing on the temperature shift from 37 °C to 30 °C was tested. Our preliminary test showed that 37 °C was suitable for cell growth while 30 °C was suitable for production. To determine the optimal timing of the shift from 37 °C to 30 °C, cultures were grown to an OD_600_ of ~0 (no culturing at 37 °C), ~0.4, or ~1 at 37 °C, then grown at 30 °C and induced as necessary. Across all strains, cultures moved to 30 °C at a later OD_600_ produced higher titers of D-psicose and grew with a greater ΔOD_600_ (Fig. 3b). When shifted at an OD_600_ ~ 1, Strain 3 produced the highest titers of D-psicose, at 9.13 g L^−1^, and grew with a ΔOD_600_ of 5.6. Strain 2 produced 5.42 g L^−1^ of D-psicose and grew with a ΔOD_600_ of 4.4.

We theorized that despite being a stronger promoter than *P*_LlacO1_, the expression timing of *P*_*gadB*_ prevents D-psicose production enzymes from siphoning carbon away from central metabolism during a key period of growth. This allows for cultures to grow more robustly and produce higher titers of D-psicose. The use of endogenous, growth phase-associated promoters eliminates the need for costly chemical inducers and allows cultures to self-regulate pathway expression depending on growth and cellular viability. *P*_*gadB*_:*alsE*-*hxpB* (pAL2247, Supplementary Table 2) was thereafter used in production experiments.

### Supplementing glucose import using GalP and Glk

Continuous glucose import, especially during the stationary phase of growth, is critical to the production of D-psicose. One consequence of limiting carbon flux through glycolysis by knocking out *pfkA* is the reduction in downstream metabolites, such as phosphoenolpyruvate (PEP)^46^. PEP is of particular concern, as it is utilized by the PTS to import and phosphorylate glucose. A reduction in PEP availability due to decreased flux through glycolysis may have an impact on the ability to assimilate glucose and produce D-psicose^46,47^. Additionally, it has been shown that increased G6P or F6P pools in a Δ*pfkA* mutant leads to degradation of *ptsG* mRNA that encodes for membrane receptor IICB^Glc^ of the PTS complex^48^.

To enhance glucose import without the use of the PTS in the Δ*pfkA* background, we additionally expressed *galP* and *glk* from a plasmid. The *galP* gene encodes for the galactose proton symporter GalP^21^, which is capable of importing glucose. The *glk* gene encodes for glucokinase Glk^22^, which phosphorylates glucose to G6P (Fig. 1c). These genes were cloned downstream of *P*_LlacO1_, generating plasmid pAL2264 (Supplementary Table 2). Induction of the expression of *galP-glk* in Strain 4 (Strain 3 with *P*_LlacO1_:*galP-glk*, Table 1) substantially increased D-psicose production and allowed the strain to produce 13.81 g L^−1^ with a specific titer of 3.2 g L^−1^ OD_600_^−1^ and yield of 55% (Fig. 3c). In comparison, Strain 4 without IPTG produced 8.67 g L^−1^ of D-psicose, with a specific titer of 1.3 g L^−1^ OD_600_^−1^ and a yield of 49%. Yield was calculated based on the possibility that for every 1 mol of D-glucose consumed, 1 mol of D-psicose could be produced, leading to a theoretical maximum yield of 100%. Interestingly, expression of *galP* and *glk* imposed a growth burden that resulted in lower ΔOD_600_ (Fig. 3c), potentially due to overexpression of membrane proteins competing for membrane translocation machinery^49^.

### Manipulating glucose utilization and metabolism

It has been shown that the PTS utilizes ~50% of PEP to transport and phosphorylate glucose^50^. To further balance cellular PEP supply and promote glucose import using GalP-Glk, we attempted to incapacitate the PTS by knocking out genes *ptsG*, encoding for membrane receptor IICB^Glc^, and *ptsH*, encoding for phosphocarrier protein HPr, in AL3990 (Supplementary Table 1)^46^.

Another source of glucose siphoning away from the D-psicose pathway is through glycogen biosynthesis. Glycogen is stored for usage during times of starvation, which for our purpose is unnecessary. The deletion of *pgm*, which encodes for phosphoglucomutase Pgm, prevents *E. coli* from producing glycogen^51^. As such, *pgm* was deleted in the production strains (Supplementary Table 1).

While Strain 4 with Δ*ptsG* appeared detrimental to psicose production (Δ*ptsG*, Δ*ptsG* Δ*ptsH* and Δ*ptsG* Δ*pgm*), Strain 4 with Δ*ptsH* has no effect (Fig. 3d). Δ*pgm* was beneficial for production, particularly when considering specific titers (Fig. 3d). Strain 5 (Strain 4 with Δ*pgm*, Table 1) produced 14.66 g L^−1^ of D-psicose, with a specific titer of 5.5 g L^−1^ OD_600_^−1^ and yield of 58% (Fig. 3d).

Inhibiting glycogen biosynthesis by knocking out *pgm* removes a source of carbon to central metabolism which would normally be used to help cells grow under periods of starvation^52^. This combined effect of impaired growth and enhanced production lead to an increase in specific titer going from Strain 4 to Strain 5 (Fig. 3d).

Although some studies have shown the elimination of the PTS through gene knockouts helps restore intracellular PEP balance and rescue growth^46^, we found knocking out *ptsG* or *ptsH* to be deleterious or neutral to D-psicose production. Phosphorylated and dephosphorylated forms of IICB^Glc^ and HPr take part in signaling cascades related not only to carbon metabolism, but also global gene expression through expression of RNA polymerase sigma subunits, including the aforementioned σ^38^-subunit and logarithmic phase-associated σ^70^-subunit^24,45,46,53,54^. Given the use of *P*_*gadB*_ to express *alsE* and *hxpB*, eliminating portions of the PTS may reduce the expression of the D-psicose production pathway genes.

### Dynamic regulation of glycolysis using CRISPRi

While the deletion of *pfkA* successfully redirected carbon flux towards D-psicose production, glycolysis remains active through PfkB. Completely shutting down glycolysis by deleting *pfkB* did not allow cells to grow under our culture conditions, so we attempted to dynamically limit *pfkB* expression to times it was essential. Cells require carbon flux through glycolysis when building biomass during the logarithmic phase of growth^42,43,55^. During the stationary phase, carbon flux can be reduced through glycolysis and redirected to D-psicose production. In order to dynamically regulate the expression of *pfkB*, we implemented a CRISPRi system^26,27^ targeting *pfkB* on the genome.

The CRISPRi system utilizes an inactivated Cas9, dCas9, which when recruited by a single guide RNA scaffold (sgRNA) can precisely target and block transcription initiation by RNA polymerase^26^. The *dcas9* gene was cloned under an anhydrotetracycline (aTc)-inducible promoter, *P*_tet_^56^, along with a constitutively expressed sgRNA sequence targeting a gene of interest. To confirm functionality of the CRISPRi system and determine where the sgRNA should target in order to achieve the greatest inhibition of expression, three different sgRNA sequences were designed to repress the expression of *sfgfp* under *P*_LlacO1_ (Supplementary Fig. 5). The sgRNAs targeted the upstream, middle, and downstream sequence of *P*_LlacO1_ (Supplementary Fig. 5a). We found the sgRNA targeting the middle of *P*_LlacO1_ lead to greatest reduction in fluorescence. Successive sgRNA targeting the promoter region of *pfkB* was designed with homology to the middle of the promoter sequence.

Further exploration of the CRISPRi system involved expressing *dcas9* and sgRNA from either the same or separate plasmids (Supplementary Fig. 6). In the single-plasmid system*, P*_tet_:*dcas9* and a constitutively expressed sgRNA sequence targeting the promoter region of *pfkB* (Supplementary Fig. 6a) or no targeting sequence were cloned onto the same plasmid (Supplementary Table 2). For the separate-plasmid system, *P*_tet_:*dcas9* was cloned onto one plasmid, and the constitutively expressed sgRNA sequence targeting the promoter region of *pfkB* or no targeting sequence were cloned onto a different plasmid (Supplementary Table 2). Each CRISPRi system was introduced into AL4186 (Supplementary Table 1), and growth was measured 24 h after induction with 100 ng mL^−1^ aTc. The separate-plasmid system caused a greater inhibition of growth, most likely because the sgRNA was expressed from a high copy number plasmid, rather than a low copy number plasmid as was the case in the single-plasmid system. When production was tested using the separate-plasmid CRISPRi system in Strain AL3990 with pAL2247 (Supplementary Table 1 and 2), very little D-psicose was produced, regardless of aTc induction. As such, the single-plasmid CRISPRi system was used for production.

**Fig. 4:**
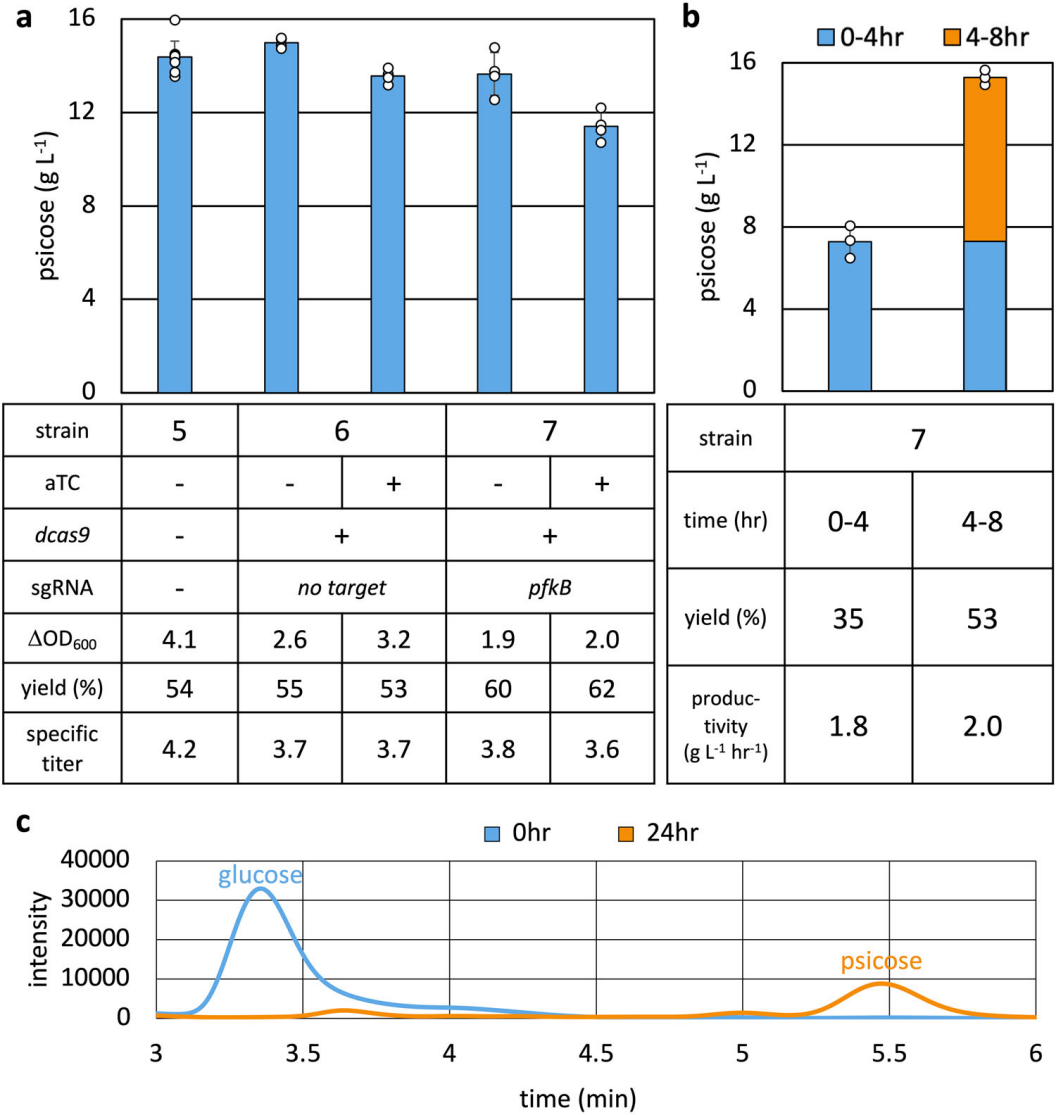
Dynamic control of glycolysis with CRISPRi. **a** CRISPRi was used to knock down *pfkB*. The sgRNA targeting the promoter region of *pfkB* or without targeting sequence was expressed from a constitutive promoter. *dcas9* was expressed from the aTc-inducible *P*_tet_. Strain 5, 6, and 7 (Table 1) were grown in M9P media with 40 g L^−1^ glucose at 37 °C to OD_600_ ~ 1, after which 1 mM IPTG and 100 ng mL^−1^ aTc were added and cells were grown at 30 ˚C for 24 h. **b** High cell density D-psicose production. Strain 7 cultures was grown in M9P media with 40 g L^−1^ glucose at 37 °C until an OD_600_ of ~1, after which they were induced with 1 mM IPTG and 100 ng mL^−^^1^ aTc and grown for another 30 min. Cultures were then spun down and resuspended in M9P media with 40 g L^−1^ glucose, 1 mM IPTG, and 100 ng mL^−1^ aTC to an OD_600_ of ~10 and grown at 30 ˚C for 8 h. Error bars indicate s.d. (*n* = 3 biological replicates). **c** HPLC chromatogram of the high cell density experiment with Strain 7 cultured for 24 h. The elution of D-glucose occurs at ~3.36 min, and the elution of D-psicose occurs at ~5.45 min.

The single-plasmid CRISPRi system was installed in Strain 5, generating Strain 6 (empty guide) and Strain 7 (sgRNA targeting *pfkB*, Table 1). When tested for D-psicose production, Strain 7 with CRISPRi showed reduced cell growth and D-psicose titer, resulting in higher specific titer in comparison to Strains 5 and 6 (Fig. 4a). Strain 7 with aTc produced 11.40 g L^−1^ of D-psicose with a specific titer of 3.6 g L^−1^ OD_600_^−1^ and yield of 62%. Strain 7 without aTc produced 13.65 g L^−1^ of D-psicose with a specific titer of 3.8 g L^−1^ OD_600_^−1^ and yield of 60%.

### D-psicose production under high culture density conditions

The rate of D-glucose consumption and D-psicose production of Strain 7 (Table 1) was monitored for 10 h at media glucose concentrations of 3, 5, and 10 g L^−1^ (Supplementary Fig. 7). We found the rate of D-glucose consumption and D-psicose production to be similar across cultures, independent of media glucose concentrations. Cultures fed 3 g L ^−1^ of glucose consumed all glucose over the course of the experiment.

To decouple growth and production, and minimize limitations on production by glucose availability, we cultured Strain 7 under high cell density conditions in an excess of available D-glucose over a shorter period of time (Fig. 4b). Strain 7 (Table 1) was grown to an OD_600_ of ~1 before being induced with 100 ng mL^−1^ aTc and 1 mM IPTG and grown for a further 30 min. Cells were then pelleted and resuspended to an OD_600_ of ~10 with M9P containing 40 g L^−1^ glucose, 100 ng mL^−1^ aTc, and 1 mM IPTG. Samples were taken and analyzed at 0, 4, and 8 h. Over the course of 8 h, Strain 7 produced 15.3 g L^−1^ of D-psicose with a specific titer of 1.4 g L^−1^ OD_600_^−1^, yield of 43%, and productivity of 1.9 g L^−1^ h^−1^ (Fig. 4b). Although D-psicose titers produced at 0–4 h and 4–8 h were similar, at 7.3 and 8.0 g L^−1^, the yield at 4–8 h (53%) was higher than that at 0–4 h (35%). The productivity at 4–8 h was 2.0 g L^−1^ h^−1^. Higher yield may be the result of cells responding to their high-density culture conditions, as our production system relies on the activation of stationary phase promoter *P*_*gadB*_. Overall, the system achieved industrially relevant production with high yield (>0.5 g_product_ g_substrate_^-1^) and high productivity (>1 g L^−1^ h^−1^) comparable to bioethanol^57^, despite being cultured in unoptimized test tube conditions.

Strain 7 was able to consume all media glucose at 24 h (Fig. 4c). Full consumption of substrate is a desirable property of microbial production, as it is costly to separate D-psicose from mixtures of glucose and/or fructose. Depleting glucose from the media allows for easier extraction and purification of D-psicose in an industrial setting.

## Conclusion

Within this study, we applied whole-cell catalysis as a strategy for producing the industrially relevant, rare sugar D-psicose. Living cells possess the capability of assembling stereo/regioselective enzymes, providing necessary cofactors, and secreting products for easy purification, all under environmentally friendly production conditions^14–17^. Whole-cell catalysis technology and infrastructure is already established industrially, and the model organism *E. coli* can be fed feedstocks that do not compete with commercial food production.

The elimination of competing pathways and additional expression of native *E*. *coli* genes (*alsE*, *hxpB*, *galP*, and *glk*) along with the use of static and dynamic gene regulation strategies lead to a strain capable of producing D-psicose using a thermodynamically favorable biosynthetic pathway from a readily available feedstock. The highest titer of D-psicose produced was 15.3 g L^−1^ with a specific titer of 2.0 g L^−1^ OD_600_^−1^ under test tube conditions. The highest yield achieved was 62%, surpassing current industrial standards. Furthermore, the strain’s ability to consume all D-glucose present in media greatly simplifies downstream purification requirements.

Overall, the engineered strain represents a critical step in producing D-psicose and other rare sugars in an efficient, cost-effective manner. The ability to produce rare sugars in bulk will help address rising global obesity rates by providing low-calorie sugar alternatives for ultra-processed foods^1,2,4^. Increased production of rare sugars will also grant access to sustainable pesticides for the agricultural industry, and medicinally relevant monosaccharides for the pharmaceutical industry^4^. The strategy developed in this study has the potential to drive transformative changes in our ability to produce, measure, and control the human-food relationship, developing a world where easily attainable metabolic health leads to happier, healthier, longer lives for everyone.

## Methods

### Reagents

All enzymes involved in the molecular cloning experiments were purchased from New England Biolabs (NEB). All synthetic oligonucleotides were synthesized by Integrated DNA Technologies. Sanger Sequencing was provided by Genewiz. D-Psicose and D-mannose were purchased from Sigma-Aldrich. D-Glucose was purchased from Fisher Scientific.

### Strains and plasmids

All strains and plasmids used in this study are listed in Supplementary Table 1 and 2, respectively. All oligonucleotides are listed in Supplementary Table 3. Plasmids for D-psicose production were constructed using sequence and ligation independent cloning^58^. The constructed plasmids were verified via sequencing. A guide to the construction of plasmids used in this study is detailed in Supplementary Table 4.

Genome modifications such as gene deletion and gene insertion were constructed using CRISPR-Cas9-mediated homologous recombination^59^. Linear DNA repair fragments for gene deletions and insertions were constructed by amplifying genomic or plasmid DNA via PCR assembly^60^. Plasmids encoding sgRNA for CRISPR-Cas9-mediated homologous recombination were constructed using Q5 site-directed mutagenesis (New England Biolabs) using pTargetF plasmid (Addgene #62226) as a template. All genomic modifications were verified via sequencing. A guide to the CRISPR-Cas9-mediated gene modifications used in this study is detailed in Supplementary Table 5.

### Predictive modeling

The Rosetta Molecular Suite was used to dock the ligand, psicose-6-phosphate, into the phosphatases: HxpA, HxpB, YigL, YidA, YihX, YbiV^37^. P6P AM1-BCC partial charges were assigned using the Antechamber suite from AMBER^61^. For the initial protein structures, we used the AlphaFold Protein Structure Database^36,62^. The AlphaFold models were then prepared for docking by using Rosetta Relax^63^. The ligand was then placed in the active site. Rosetta GALigand Dock was then used to dock P6P conformers into each phosphatase^64^. Distance and angle constraints were integrated to maintain the D-psicose and magnesium ion in catalytically competent geometry for hydrolysis. A total of 2000 simulations were run for each phosphatase, and the top 10 best scoring outputs sorted by constraint score, protein-ligand interface energy, and total system energy score were selected for analysis.

### Culturing media

Overnight cultures were grown at 37 °C in 3 mL of Luria-Bertani (LB) media with appropriate antibiotics. Antibiotic concentrations were as follows: spectinomycin (50 μg mL^−1^), ampicillin (200 μg mLSLIC^1^), kanamycin (50 μg mLSLIC^1^), gentamycin (3.75 μg mL^−^^1^). M9 minimal media consists of 33.7 mM Na_2_HPO_4_, 22 mM KH_2_PO_4_, 8.6 mM NaCl, 9.4 mM NH_4_Cl, 2 mM MgSO_4_, 0.1 mM CaCl_2_, A5 trace metals mix (2.86 mg L^−^^1^ H_3_BO_3_, 1.81 mg L^−1^ MnCl_2_⋅4H_2_O, 0.079 mg L^−1^ CuSO_4_⋅5H_2_O, 49.4 μg L^−1^ Co(NO_3_)_2_⋅6H_2_O), varying concentrations of glucose, and appropriate antibiotics. M9P media for the psicose production consists of M9 minimal media supplemented with 5 g L^−^^1^ of yeast extract and appropriate antibiotics. No D-psicose production was detected when cultures were grown in M9P media without glucose. Inducer concentrations are as follows: isopropyl-β-D-1-thiogalactopyranoside (IPTG) (1 mM), anhydrotetracycline (aTc) (100 ng mL^−1^). OD_600_ was measured with a Synergy H1 hybrid plate reader (BioTek Instruments, Inc.).

### Fluorescence assays

Overnight cultures were inoculated at 1% into 300 μL of LB media in a 96-well black-walled fluorescence assay plate. Cells were grown at 37 °C, 250 rpm, until OD_600_ ~ 0.4. Cultures were then induced with IPTG if necessary and grown at 37 °C, 250 rpm, for 24 h. Fluorescence was measured at an excitation wavelength of 485 nm and emission wavelength of 510 nm with a Synergy H1 hybrid plate reader (BioTek Instruments, Inc.).

### D-Psicose production

For regular cell density production experiments, overnight cultures were inoculated at 1% into 3 mL of M9P media. Cells were grown at 37 °C until the OD_600_ described, then induced with IPTG and aTc if necessary, and grown at 30 °C for 24 h.

For high cell density production experiments in M9P media, overnight cultures were inoculated at 2% into 50 mL of M9P media. Cells were grown at 37 °C until OD_600_ ~ 1. Cultures were then induced with IPTG and aTc if necessary and grown for a further 30 min. Cultures were centrifuged at 5000 g for 15 min and resuspended in M9P media with IPTG and aTc if necessary to a target OD_600_. Cultures were grown at 30 °C for 24 h.

### HPLC analysis

Analysis of D-psicose, D-glucose, and D-mannose concentrations was performed using HPLC (Shimadzu) equipped with a refractive index detector (RID) 10 A and Rezex™ RCU-USP sugar alcohol column (Phenomenex). Mobile phase was comprised of 100% MilliQ water. Samples were run with an injection volume of 1 μL at a flow rate of 0.5 mL min^−^^1^ for 7 min, with the column oven at 83 °C and RID cell temperature of 40 °C.

To prepare samples for HPLC analysis, 300 μL of culture was centrifuged at 17,000 g for 5 min. Supernatants were applied to a 0.2 μm PVDF hydrophilic membrane 96 well filter plate and centrifuged at 17,000 g for 2 min into a polystyrene 96 well.

### GC-MS analysis

GC-MS analysis was performed by the UC Davis West Coast Metabolomics Center. Chemical standards (D-psicose, D-mannose, D-glucose, D-galactose, D-erythrose, D-tagatose, and D-threose) were purchased from Sigma Aldrich.

To analyze via GC-MS, 4 μL of spun-down culture supernatant were dried down and derivatized by adding 10 μL of 40 mg mL^−^^1^ methoxyamine hydrochloride (Sigma-Aldrich) in pyridine (Sigma-Aldrich) and shaking at 30 °C for 1.5 h. Subsequently, 90 μL of *N* –methyl-N-(trimethylsilyl)-trifluoroacetamide (MSTFA) (Sigma-Aldrich) was added with a standard mixture of 13 fatty-acid methyl esters (FAMEs) (Sigma-Aldrich) as retention index markers and shaken at 80°C for 30 min. Samples were immediately transferred to crimp top vials and injected onto each GC − MS instrument. A LECO Pegasus IV TOF MS was coupled to an Agilent 7890 GC system installed with a Restek RTX-5Sil MS column (29.70 m length, 0.25 mm i.d, 0.25 μM df, 95% dimethyl/ 5% diphenyl polysiloxane film) with an additional 10 m guard column. 1 μL of the derivatized sample was injected into the GC in splitless mode at an injection temperature of 275 °C and a constant flow of 1 mL min^−1^. The initial oven temperature was held at 50 °C for 1 min and ramped at a rate of 20 °C min^−1^ to 330 °C that was maintained for 5 min for a total run time of 20 min. The mass spectrometer was used under electron ionization mode at +70 eV. Mass spectra were acquired from 85 to 500 m/z at a scan rate of 17 Hz and 250 °C source temperature. Binbase was used for metabolite annotation and reporting^65^.

### Supplementary information

Supplementary Information

nr-reporting-summary

## Data Availability

The datasets generated in this study are available from the corresponding author on request. The *E. coli* strains and the plasmids used in this study are available upon request.
