# Supplementary material for: Awakening the natural capability of psicose production in Escherichia coli
Source: NPJ Sci Food. 2023 Oct 14;7:54. doi: 10.1038/s41538-023-00231-0 (PMC10576766; doi:10.1038/s41538-023-00231-0)
Supplement: Supplementary file 1 — Supplementary Information [file 41538_2023_231_MOESM1_ESM.pdf]

**Supplementary Information**

**Awakening the natural capability of psicose production in *Escherichia coli***

Jayce E. Taylor[1], Dileep Sai Kumar Palur[1], Angela Zhang[1], Jake N. Gonzales[2], Augustine Arredondo[1], Timothy A. Coulther[3], Amiruddin Bin Johan Lechner[1], Elys P. Rodriguez[1,4], Oliver Fiehn[4], John Didzbalis[5], Justin B. Siegel[1,3,6], and Shota Atsumi[1,2*]

[1]Department of Chemistry, University of California, Davis, Davis, CA, 95616, USA

[2]Plant Biology Graduate Group, University of California, Davis, Davis, CA, 95616, USA

[3]Genome Center, University of California, Davis, Davis, CA 95616, USA.

[4]West Coast Metabolomics Center, UC Davis Genome Center, University of California, Davis, Davis, CA 95616

[5]Mars, Incorporated, 6885 Elm Street, McLean, VA 22101, USA

[6]Department of Biochemistry and Molecular Medicine, University of California, Davis, Sacramento, CA 95616, USA.

*To whom correspondence may be addressed: E-mail: satsumi@ucdavis.edu

**Supplementary Table 1 Strains used in this study**

| Strain | Genotype | Source |
|---|---|---|
| MG1655 | F- lambda- *ilvG- rfb*-50 *rph*-1 | Ref. 1 |
| AL1050 | MG1655, but with attB::*$lacI^q$ tetR* $spec^R$ | Ref. 2 |
| AL3601 | AL1050, but with ss9::$P_{lacUV5}$:*T7rnap* | Ref. 3 |
| AL4058 | MG1655, but with Δ*pfkA* | This study |
| AL3694 | AL3601, but with Δ*pfkA* | This study |
| AL4063 | AL3694, but with Δ*alsE* | This study |
| AL4082 | AL4058, but with Δ*alsE* | This study |
| AL3756 | AL1050, but with Δ*pfkA* Δ*zwf* Δ*rpiB* | This study |
| AL3990 | AL3756, but with Δ*manA* | This study |
| AL4121 | AL3990, but with Δ*ptsG* | This study |
| AL4188 | AL3990, but with Δ*ptsH* | This study |
| AL4189 | AL4121, but with Δ*ptsH* | This study |
| AL4186 | AL3990, but with Δ*pgm* | This study |
| AL4187 | AL4121, but with Δ*pgm* | This study |
| AL4200 | AL4188, but with Δ*pgm* | This study |
| AL4201 | AL4189, but with Δ*pgm* | This study |

**Supplementary Table 2 Plasmids used in this study**

| Plasmid | Description | Source |
|---|---|---|
| pCas | $P_{\mathrm{cas}}$:*cas9* $P_{araB}$:*red lacI*$^{q}$ $P_{\mathrm{trc}}$:sgRNA pMB1 repA101(Ts) kan$^{R}$ | addgene #62225 |
| pTargetF | $P_{\mathrm{J23119}}$:sgRNA-*pMB1*, spec$^{R}$, pMB1 | addgene #62226 |
| pAL421 | $P_{\mathrm{LlacO1}}$:*sfgfp*, amp$^{R}$, ColE1 | Ref. 3 |
| pAL1494 | $P_{\mathrm{T7}}$, amp$^{R}$, ColE1 | This study |
| pAL1759 | $P_{\mathrm{LlacO1}}$, amp$^{R}$, ColE1 | Ref. 3 |
| pAL1851 | pTargetF-*lacZ*, amp$^{R}$, ColE1 | Ref. 3 |
| pAL1874 | $P_{\mathrm{tet}}$:*dcas9*, cm$^{R}$, p15A | addgene #44249 |
| pAL1946 | $P_{\mathrm{T7}}$:*alsE-hxpB*, amp$^{R}$, ColE1 | This study |
| pAL1947 | $P_{\mathrm{T7}}$:*alsE-ybiV*, amp$^{R}$, ColE1 | This study |
| pAL1950 | pTargetF-*pfkA*, amp$^{R}$, ColE1 | This study |
| pAL1952 | $P_{\mathrm{tet}}$:*dcas9*, kan$^{R}$, p15A | This study |
| pAL1957 | pTargetF-*rpiB*, amp$^{R}$, ColE1 | This study |
| pAL1958 | pTargetF-*zwf*, amp$^{R}$, ColE1 | This study |
| pAL1974 | $P_{ihfA4}$:*sfgfp*, amp$^{R}$, ColE1 | This study |
| pAL1975 | $P_{dps}$:*sfgfp*, amp$^{R}$, ColE1 | This study |
| pAL1976 | $P_{cbpA2}$:*sfsfp*, amp$^{R}$, ColE1 | This study |
| pAL1977 | $P_{gadB}$:*sfgfp*, amp$^{R}$, ColE1 | This study |
| pAL2001 | $P_{\mathrm{LlacO1}}$:*alsE-hxpB*, amp$^{R}$, ColE1 | This study |
| pAL2036 | pTargetF-*ptsG*, amp$^{R}$, ColE1 | This study |
| pAL2038 | pTargetF-*pgm*, amp$^{R}$, ColE1 | This study |
| pAL2062 | $P_{\mathrm{LlacO1}}$:*sfgfp*, pTargetF-*pfkA*, amp$^{R}$, ColE1 | This study |
| pAL2063 | $P_{\mathrm{LlacO1}}$:*sfgfp*, pTargetF-no target, amp$^{R}$, ColE1 | This study |
| pAL2066 | $P_{\mathrm{LlacO1}}$:*sfgfp*, pTargetF-sgRNA A*, amp$^{R}$, ColE1 | This study |
| pAL2124 | pTargetF-*pfkB*, amp$^{R}$, ColE1 | This study |
| pAL2158 | pTargetF-no target, amp$^{R}$, ColE1 | This study |
| pAL2173 | $P_{\mathrm{LlacO1}}$:*sfgfp*, pTargetF-sgRNA B*, amp$^{R}$, ColE1 | This study |
| pAL2174 | $P_{\mathrm{LlacO1}}$:*sfgfp*, pTargetF-sgRNA C*, amp$^{R}$, ColE1 | This study |
| pAL2178 | pTargetF-*manA*, amp$^{R}$, ColE1 | This study |
| pAL2182 | $P_{\mathrm{tet}}$:*dcas9*, pTargetF-no target, kan$^{R}$, p15A | This study |
| pAL2188 | $P_{\mathrm{tet}}$:*dcas9*, pTargetF-*pfkB*, kan$^{R}$, p15A | This study |
| pAL2233 | pTargetF-*alsE*, amp$^{R}$, ColE1 | This study |
| pAL2247 | $P_{gadB}$:*alsE-hxpB*, amp$^{R}$, ColE1 | This study |
| pAL2256 | $P_{\mathrm{LlacO1}}$:*galP-glk*, kan$^{R}$, ColA | This study |
| pAL2264 | $P_{\mathrm{LlacO1}}$:*galP-glk*, gent$^{R}$, ColA | This study |
| pAL2318 | pTargetF-*ptsH*, amp$^{R}$, ColE1 | This study |
| pAL2348 | $P_{\mathrm{T7}}$:*alsE-hxpA*, amp$^{R}$, ColE1 | This study |
| pAL2349 | $P_{\mathrm{T7}}$:*alsE-yigL*, amp$^{R}$, ColE1 | This study |

| | | |
|---|---|---|
| pAL2351 | $P_{T7}$:*alsE-yidA*, amp$^R$, ColE1 | This study |
| pAL2352 | $P_{T7}$:*alsE-yihX*, amp$^R$, ColE1 | This study |

* See Supplementary Figure 5.

**Supplementary Table 3 Oligonucleotides used in this study**

| Name | Sequence 5' to 3' | Plasmid(s) or fragment(s) produced |
|---|---|---|
| AZ0195 | GCACATCAGCGTTTTAGAGCTAGAAATAGC | pAL2173 |
| AZ0196 | TCAGTATCTTACTAGTATTATACCTAGGAC | pAL2173 |
| AZ0466 | GCCATATCGAAGGTCGTCATATGAAAATCTCCCCCTCGTTAATG | pAL1946, pAL1947 |
| AZ0467 | ATCTCCTTTTGTTAGCAGCCGGATCCTTATGCTGTTTTTGCATGAGGCTG | pAL1946, pAL1947 |
| AZ0468 | GGCTGCTAACAAAAGGAGATATACATATGTCAACCCCGCGTCAGATTCTTGC | pAL1946 |
| AZ0469 | GCTTTGTTAGCAGCCGGATCCTCAACCGAGAAGGTCTTTTGCGGTG | pAL1946 |
| AZ0472 | GGCTGCTAACAAAAGGAGATATACATATGAGCGTAAAAGTTATCGTCAC | pAL1947 |
| AZ0473 | GCTTTGTTAGCAGCCGGATCCTCAGCTGTTAAAAGGGGATGTG | pAL1947 |
| AZ0475 | GGATCCGGCTGCTAACAAAGCCCGAAAGGAAGCTGAGTTGGC | pAL1946, pAL1947 |
| AZ0482 | CTGACCTGAATCAATTCAGCAGGAAGTGATTGTTATACTATTTGCACATTCGTTGGAT | Δ*pfkA* repair fragment (RF) |
| AZ0483 | TCTGTTGCCGGAAGTCTTCTTGCACATCGAAGTGATCCAACGAATGTGCAAATAGTAT | Δ*pfkA* RF |
| AZ0484 | AGACTTCCGGCAACAGATTTCATTTTGCATTCCAAAGTTCAGAGGTAGTCTGATTTCG | Δ*pfkA* RF |
| AZ0485 | TGTCATCGGTTTCAGGGTAAAGGAATCTGCCTTTTTCCGAAATCAGACTACCTCTGAAC | Δ*pfkA* RF |
| AZ0486 | ACCCTGAAACCGATGACAGAAGCAAAAATGCCTGATGCGCTTCGCTTATCAGGCCTACAT | Δ*pfkA* RF |
| AZ0487 | CCTACAAAAGTTTGCAAATTCAATAAATTGCAGAATTCATGTAGGCCTGATAAGCGA | Δ*pfkA* RF |
| AZ0502 | CAAGCGAGCTCGATATCAAATCAGAAGAACTCGTCAAGAAGGC | pAL1952 |
| AZ0503 | CAGGAGCTAAGGAAGCTAAAATGATTGAACAAGATGGATTGCACGCAGG | pAL1952 |
| AZ0504 | TTTAGCTTCCTTAGCTCCTGAAAATCTCGATAACTC | pAL1952 |
| AZ0505 | TTTGATATCGAGCTCGCTTGGACTCC | pAL1952 |
| AZ0520 | TCGGTCTGCCGTTTTAGAGCTAGAAATAGC | pAL1950 |
| AZ0521 | TGCACGGGAAACTAGTATTATACCTAGGAC | pAL1950 |
| AZ0522 | CTGTTGCTGGGTTTTAGAGCTAGAAATAGC | pAL1957 |
| AZ0523 | CCAGTGCGACACTAGTATTATACCTAGGAC | pAL1957 |
| AZ0526 | CGCTGTCAGATGTAACTCTGTAAAACAGATCAGGAAGGCGTA | Δ*rpiB* RF |
| AZ0527 | TGTGATGTTAATGAATTAAAAACACCCAAAATCGATGAATTACGCCTTCCTGATCTGTT | Δ*rpiB* RF |
| AZ0528 | GGGTGTTTTTAATTCATTAACATCACAAATGTTTTTTGATTGTGAAGTTTTGCACGGACG | Δ*rpiB* RF |
| AZ0529 | CTCATCCATGCAAGTAGTGGATGAATCTCATCTTCCCCGTCCGTGCAAAACTTCACAAT | Δ*rpiB* RF |
| AZ0530 | CCACTACTTGCATGGATGAGTAATGATTAATGTGGATAGAGTTTCTTTTTGAGGTTGGCT | Δ*rpiB* RF |
| AZ0531 | AGCGGAAAGCGTTTCATTAGCCAACCTCAAAAAGAAACTCTA | Δ*rpiB* RF |
| AZ0532 | ATGAAACGCTTTCCGCTATTTCTTTTATTCACCCTGCTCACGCTGTCCACCGTTC | Δ*rpiB* RF |
| AZ0533 | CGGAACGGTGGACAGCGTGAGC | Δ*rpiB* RF |
| AZ0534 | TGGGGATCGAGTTTTAGAGCTAGAAATAGC | pAL1958 |
| AZ0535 | CTTCTTCTGCACTAGTATTATACCTAGGAC | pAL1958 |

| | | |
|---|---|---|
| AZ0538 | TAATCGCACGGGTGGATAAGCGTTTACAGTTTTCGCAAGCTCGTAAAAGCAGTACAGTGC | Δ*zwf* RF |
| AZ0539 | CGGTACTTAAGCCAGGGTATACTTGTAATTTTCTTACGGTGCACTGTACTGCTTTTACGA | Δ*zwf* RF |
| AZ0540 | ACCCTGGCTTAAGTACCGGGTTAGTTAACTTAAGGAGAATGACTATCTGCGCTTATCCT | Δ*zwf* RF |
| AZ0541 | GCGCAAGATCATGTTACCGGTAAAATAACCATAAAGGATAAGCGCAGATAGTCATT | Δ*zwf* RF |
| AZ0542 | CGGTAACATGATCTTGCGCAGATTGTAGAACAATTTTTACACTTTCAGGCCTCGTGCGGA | Δ*zwf* RF |
| AZ0543 | CAGTCAGTGTAATAAAAAAAGCCTCGTGGGTGAATCCGCACGAGGCCTGAA | Δ*zwf* RF |
| AZ0666 | AAGAGGAGAAAAGATATACCATGAAAATCTCCCCCTCGTTAATGTG | pAL2001, pAL2247 |
| AZ0667 | GGTACCTTAGCAGCCGGATCTCAACCGAGAAGGTCTTTT | pAL2001, pAL2247 |
| AZ0668 | GATCCGGCTGCTAAGGTACCTAATCTAGAGGCATC | pAL2001, pAL2247 |
| AZ0669 | GGTATATCTTTTCTCCTCTTTAATGAATTCGGTCAGTGCG | pAL2001, pAL2247 |
| AZ0711 | ACAATCGTGCGTTTTAGAGCTAGAAATAGC | pAL2038 |
| AZ0712 | GGATTGCCATACTAGTATTATACCTAGGAC | pAL2038 |
| AZ0743 | CTGAATTATTTTACTCTGTGTAATAAATAAAGGGCGCTTAGATGCCCTGTACACGGCGA | Δ*ptsG* RF |
| AZ0744 | ACGCGTGGCAAGGGGGGAGAGCCTCGCCGTGTACAGGGCATCTAAGCG | Δ*ptsG* RF |
| AZ0745 | CCTTGCCACGCGTGAGAACGTAAAAAAAGCACCCATACTCAGGAGCACTCTCAATTTCCG | Δ*ptsG* RF |
| AZ0746 | GCCATCTGGCTGCCTTAGTCTCCCCAACGTCTTACGGAAATTGAGAGTGCTCCT | Δ*ptsG* RF |
| AZ0747 | GGCAGCCAGATGGCTGCCTTTTTTACAGGTGTTATTCAGAATTGATACGTGCCGGTAATG | Δ*ptsG* RF |
| AZ0748 | GTGGAAGGTTCTATCGTCTACGGCACACCGCGTAATTTCAGCATTACCGGCACGTATCAA | Δ*ptsG* RF |
| AZ0751 | AACCTGCAAAGTTTTAGAGCTAGAAATAGC | pAL2036 |
| AZ0752 | AGCAAATGCAACTAGTATTATACCTAGGAC | pAL2036 |
| AZ0763 | CAACACCAACACCGGCCGTAAATGCAGCATGATCGAACACATCATGCAGTC | Δ*pgm* RF |
| AZ0764 | TCCGCAAACCTTCTCAATCAATTCCGCCGGGAATTGCATCGACTGCATGATGTGTTCGA | Δ*pgm* RF |
| AZ0765 | GATTGAGAAGGTTTGCGGAACTATCTAAAACGTTGCAGACAAAGGACAAAGCAACACATT | Δ*pgm* RF |
| AZ0766 | AAGGGCGATCTTGCGACCGCCCTTTTTTTATTAAATGTGTTGCTTTGTCCTTTGTCT | Δ*pgm* RF |
| AZ0767 | CGCAAGATCGCCCTTTTTTACGTATGACAAACACAGAATTGCCTGATGCGCTACGCTTAT | Δ*pgm* RF |
| AZ0768 | TCGCTTAAATTCAATATATTGCACCATCCTCGTAGGCCTGATAAGCGTAGCGCATCAG | Δ*pgm* RF |
| AZ0936 | ATGACGACCTTCGATATGGCCGCTGCTGTG | pAL1946, pAL1947 |
| AZ1025 | AAATTGTGAGGTTTTAGAGCTAGAAATAGC | pAL2174 |
| AZ1026 | AATAGATCTAACTAGTATTATACCTAGGAC | pAL2174 |
| AZ1041 | AATATCCTACACACTTTTTTAACAAAAACTGAGACTAGTACGACTTTTTGCGGCTCC | Δ*manA* RF |
| AZ1042 | CCCACTATTAAAGCAAGAATCCTACGGGAAGTAACCTGGAGCCGCAAAAAGTCG | Δ*manA* RF |
| AZ1043 | AGGATTCTTGCTTTAATAGTGGGATTAATTTCCACATTAAAACAGGGATTGATCGAGCT | Δ*manA* RF |
| AZ1044 | GCCTTTAATAAGCTTAGCAAGAGATGTTAATTTTTTCAGTAAGCTCGATCAATCCCTGT | Δ*manA* RF |
| AZ1045 | TCTCTTGCTAAGCTTATTAAAGGCTTATAACACCTTCAGGCGGCCAGTCCGCCTGATTTC | Δ*manA* RF |
| AZ1046 | CTACCGCTACCAGCGATTTATTCATAATGATTATCCATAAAATGAAATCAGGCGGACTGG | Δ*manA* RF |
| AZ1051 | TCCTCGCTCACTGACTCGCTTCCTTACGCATCTGTGCGGTATTTCACACC | pAL2182 |
| AZ1052 | GCAGTCGAACGACCGAGCGTGGATAACAGGGTAATAGATCTAAGCTTCTG | pAL2182 |
| AZ1053 | ACGCTCGGTCGTTCGACTGCGG | pAL2182 |
| AZ1054 | AGCGAGTCAGTGAGCGAGGAAGCGGAATATATCC | pAL2182 |

| | | |
|---|---|---|
| AZ1055 | GTTTTAGAGCTAGAAATAGCAAG | pAL2063, pAL2158 |
| DS071 | TCATGACGGCACTAGTATTATACCTAGGACTGAGCTAG | pAL2233 |
| DS072 | CATGAAAGTGGTTTTAGAGCTAGAAATAGCAAGTTAAAATAAG | pAL2233 |
| DS075 | CGCCATCATTGGCGGCACCAGTTTCTTCGGCGGCAAGGGGCGCATTTTCTCT | Δ*alsE* RF |
| DS076 | GTTGTTGATGGTGCCGATGATCAACCCGCCAATCACCACAGAGAAAATGCGCCCCT | Δ*alsE* RF |
| DS077 | TCGGCACCATCAACAACGGTCTGAATATTTTGCAGGTACAAACCTATTACCAACTGGTGG | Δ*alsE* RF |
| DS078 | CAAGGGCGACAGCCGCGATAATTAATCCGCCCATCACCACCAGTTGGTAATAGGTTTG | Δ*alsE* RF |
| DS079 | GGCTGTCGCCCTTGACCGTCTTATCAGTAAGTAAGGAATTGAACCGTCGTAGCGGGCGTG | Δ*alsE* RF |
| DS080 | TGCTGTCCGCAGACAAAAGCGGATATGCGTTGCCCCCATATCCACGCCCGCTACG | Δ*alsE* RF |
| DS081 | TGTCTGCGGACAGCAGAAGGTGAAACGCTACACTGCGAAAAAAAGCGGACCGCAGAAGTC | Δ*alsE* RF |
| DS082 | AGGCCGGGAGCAATGACTTCTGCGGTCCGCTTTTTTTCGCAGTG | Δ*alsE* RF |
| DS083 | TTGCTCCCGGCCTGGTGTCGGGTATCGGCGAAATGATTGACGAGCAACTCAGGCGCTTTA | Δ*alsE* RF |
| DS084 | CCGGAAATCCCATCACCAGACCATGACAGCGAGCGTTAAAGCGCCTGAGTTGCTCGT | Δ*alsE* RF |
| DS126 | TGAGCGGGACTCTGGGGTTCGAG | pAL2264 |
| DS127 | GCGAAACGATCCTCATCCTGTCTCTTG | pAL2264 |
| DS128 | TGAGGATCGTTTCGCATGTTACGCAGCAGCAACGATG | pAL2264 |
| DS129 | CCAGAGTCCCGCTCATTAGGTGGCGGTACTTGGGTC | pAL2264 |
| DS157 | GTTTAAACAGACTAGTATTATACCTAGGAC | pAL2318 |
| DS158 | TGCAGACTCTGTTTTAGAGCTAGAAATAGC | pAL2318 |
| DS159 | GGACTGTATTGCGCTCTTCGTGCGTCGCGTCTGTTAA | Δ*ptsH* RF |
| DS160 | GCCTGGCGGTTCGACTTTAGCCTGTATTGTTAGCGCCAGTTTTTAACAGACGCGACGCAC | Δ*ptsH* RF |
| DS161 | CGAACCGCCAGGCTAGACTTTAGTTCCACAACACTAAACCTAT | Δ*ptsH* RF |
| DS162 | CCGGGAAATGTATTTCCCCAACTTATAGGTTTAGTGTTGTGGAACTAAAGTCTA | Δ*ptsH* RF |
| DS163 | GGGGAAATACATTTCCCGGGTTCTTTTAAAAATCAGTCACAAGTAAGGTAGGGTTATGA | Δ*ptsH* RF |
| DS164 | CCGAAAGCGATACCCGGGGATGCTAAAATGCCTGAAATCATAACCCTACCTTACTTGTGA | Δ*ptsH* RF |
| DS165 | CGGGTATCGCTTTCGGTAAAGCTCTGCTTCTGAAAGAAGACGAAATTGTCATTGACCGGA | Δ*ptsH* RF |
| DS166 | CCTGGTCGGCAGAAATTTTTTTCCGGTCAATGACAATTTCGTCTT | Δ*ptsH* RF |
| DS303 | CGGATCCATCTCCTTTTGTTAGCAG | pAL2348, pAL2349, pAL2351, pAL2352 |
| DS304 | GCTGCTAACAAAGCCCGAAAGGAAG | pAL2348, pAL2349, pAL2351, pAL2352 |
| DS305 | AACAAAAGGAGATGGATCCGGTGCGGTGCAAAGGTTTTCTG | pAL2348 |
| DS306 | TTTCGGGCTTTGTTAGCAGCTCACTGAATAATAACATCGC | pAL2348 |
| DS307 | AACAAAAGGAGATGGATCCGATGTACCAGGTTGTTGCGTC | pAL2349 |
| DS308 | TTTCGGGCTTTGTTAGCAGCTTACGATAAATAGAGTTTAC | pAL2349 |
| DS311 | AACAAAAGGAGATGGATCCGATGGCTATTAAACTCATTGC | pAL2351 |
| DS312 | TTTCGGGCTTTGTTAGCAGCTTAATTCAGCACATACTTCT | pAL2351 |
| DS313 | AACAAAAGGAGATGGATCCGATGCTCTATATCTTTGATTTAG | pAL2352 |
| DS314 | TTTCGGGCTTTGTTAGCAGCTTAGCATAACACCTTCGCG | pAL2352 |

| | | |
|---|---|---|
| JET003 | CTCGAGGTGAAGACGAAAGGGCCTCGTGATACG | pAL1974, pAL1975, pAL1976, pAL1977 |
| JET004 | CGAATTCATTAAAGAGGAGAAAGGTACCATGGGTCATCACC | pAL1974, pAL1975, pAL1976, pAL1977 |
| JET005 | TTCGTCTTCACCTCGAGTATCCGAATGTAAGAAAGTTGGC | pAL1974 |
| JET006 | CCTCTTTAATGAATTCGAGGTTCAATCCCTCAATGATGCC | pAL1974 |
| JET007 | CGTCTTCACCTCGAGTCATTGAATCTTTATTAGTTTTGTTTTTCACGC | pAL1975 |
| JET008 | CCTCTTTAATGAATTCGAATTTCATATCCTCTTGATGTTATGTCCC | pAL1975 |
| JET009 | CACCTCGAGTTTGCAGTGCAACTAATTCC | pAL1976 |
| JET010 | CCTCTTTAATGAATTCGAGCGTTATCTCGC | pAL1976 |
| JET011 | GCCCTTTCGTCTTCACCTCGAGGTAATAATTTTATAAATGCG | pAL1977, pAL2247 |
| JET012 | CCTCTTTAATGAATTCGACTTGCTTCTTATCCATTTTAAACTCC | pAL1977, pAL2247 |
| JET018 | AAGAGGAGAAAAGATATACCATGAAAATCTCCCCCTCGTTAATGTG | pAL2247 |
| JET067 | ATAGCCAGCAGTTTTAGAGCTAGAAATAGC | pAL2178 |
| JET068 | CGAGGGCCGAACTAGTATTATACCTAGGAC | pAL2178 |
| JET087 | TCCGCTGATTGTTTTAGAGCTAGAAATAGC | pAL2124 |
| JET087 | TCCGCTGATTGTTTTAGAGCTAGAAATAGC | pAL2188 |
| JET088 | AAGTGAAGAAACTAGTATTATACCTAGGACTGAGC | pAL2124 |
| JET088 | AAGTGAAGAAACTAGTATTATACCTAGGACTGAGC | pAL2188 |
| JET134 | GGTATATCTTTTCTCCTCTTTAATGAATTCACTTGCTTCTTATCCATTTTAAACTCC | pAL2247 |
| JG190 | GAAAGGTACCGTTTTAGAGCTAGAAATAGC | pAL2066 |
| JG191 | TCCTCTTTAAACTAGTATTATACCTAGGAC | pAL2066 |
| MM57 | ACTAGTATTATACCTAGGACTGAGC | pAL2063, pAL2158 |

**Supplementary Table 4 Plasmid construction guide**

| | Vector PCR | | | Insert(s) PCR | | | |
|---|---|---|---|---|---|---|---|
| Plasmid | Primer (F) | Primer (R) | Template | Primer (F) | Primer (R) | Template | Insert description |
| pAL1946 | AZ0475 | AZ936 | pAL1494 | AZ0466 | AZ0467 | MG1655 | *alsE* |
| | | | | AZ0468 | AZ0469 | MG1655 | *hxpB* |
| pAL1947 | AZ0475 | AZ936 | pAL1494 | AZ0466 | AZ0467 | MG1655 | *alsE* |
| | | | | AZ0472 | AZ0473 | MG1655 | *ybiV* |
| pAL1950* | AZ0520 | AZ0521 | pAL1851 | | | | |
| pAL1952 | AZ0504 | AZ0505 | pAL1874 | AZ0502 | AZ0503 | pAL1949 | *kanR* |
| pAL1957* | AZ0523 | AZ0522 | pAL1851 | | | | |
| pAL1958* | AZ0534 | AZ0535 | pAL1851 | | | | |
| pAL1974 | JET004 | JET003 | pAL421 | JET005 | JET006 | MG1655 | $P_{ihfA4}$ |
| pAL1975 | JET004 | JET003 | pAL421 | JET007 | JET008 | MG1655 | $P_{dps}$ |
| pAL1976 | JET004 | JET003 | pAL421 | JET009 | JET010 | MG1655 | $P_{cbpA2}$ |
| pAL1977 | JET004 | JET003 | pAL421 | JET011 | JET012 | MG1655 | $P_{gadB}$ |
| pAL2001 | AZ0668 | pAL669 | pAL1759 | AZ0666 | AZ0667 | pAL1946 | *alsE-hxpB* |
| pAL2036* | AZ0751 | AZ0752 | pAL1851 | | | | |
| pAL2038* | AZ0771 | AZ0712 | pAL1851 | | | | |
| pAL2063* | AZ1055 | MMM57 | pAL2062 | | | | |
| pAL2066* | JG190 | JG191 | pAL2062 | | | | |
| pAL2124* | JET087 | JET088 | pAL1851 | | | | |
| pAL2158* | AZ1055 | MMM57 | pAL1851 | | | | |
| pAL2173* | AZ195 | AZ196 | pAL2066 | | | | |
| pAL2174* | AZ1025 | AZ1016 | pAL2066 | | | | |
| pAL2178 * | JET167 | JET168 | pAL1851 | | | | |
| pAL2182 | AZ1053 | AZ1054 | pAL1952 | AZ1051 | AZ1052 | pAL2063 | pTargetF- *no target* |
| pAL2188* | JET087 | JET088 | pAL2182 | | | | |
| pAL2233* | DS072 | DS071 | pAL1851 | | | | |
| pAL2247 | AZ0666 | JET018 | pAL2001 | JET011 | JET134 | pAL1977 | $P_{gadB}$ |
| pAL2264 | DS127 | DS126 | pAL2256 | DS129 | DS128 | pAL2257 | $gent^R$ |
| pAL2318* | DS158 | DS157 | pAL1851 | | | | |
| pAL2348 | DS304 | DS303 | pAL1946 | DS305 | DS306 | genome | *hxpA* |
| pAL2349 | DS304 | DS303 | pAL1946 | DS307 | DS308 | genome | *yigL* |
| pAL2351 | DS304 | DS303 | pAL1946 | DS311 | DS312 | genome | *yidA* |
| pAL2352 | DS304 | DS303 | pAL1946 | DS313 | DS314 | genome | *yihx* |

*Q5-site directed mutagenesis (NEB)

**Supplementary Table 5 Guide for CRISPR-Cas9-mediate gene deletions and insertions**

| Modification | pTargetF | | Linear repair fragment assembly PCR primers |
|---|---|---|---|
| | Plasmid | 20 bp sgRNA targeting sequence 5' to 3' | |
| Δ*pfkA* | pAL1950 | TTCCCGTGCATCGGTCTGCC | AZ0482(F), AZ0483(R), AZ0484(F), AZ0485(R), AZ0486(F), AZ0487(R) |
| Δ*alsE* | pAL2233 | GCCGTCATGACATGAAAGTG | DS075(F), DS076(R), DS078(F), DS079(R), DS080(F), DS081(R), DS082(F), DS083(R) |
| Δ*zwf* | pAL1958 | GCAGAAGAAGTGGGGATCGA | AZ0538(F), AZ0539(R), AZ0540(F), AZ0541(R), AZ0542(F), AZ0543(R) |
| Δ*rpiB* | pAL1957 | GTCGCACTGGCTGTTGCTGG | AZ0526(F), AZ0527(R), AZ0528(F), AZ0529(R), AZ0530(F), AZ0531(R), AZ0532(F), AZ0533(R) |
| Δ*manA* | pAL2178 | TCGGCCCTCGATAGCCAGCA | AZ1041(F), AZ1042(R), AZ1043(F), AZ1044(R), AZ1045(F), AZ1046(R) |
| Δ*ptsG* | pAL2036 | TGCATTTGCTAACCTGCAAA | AZ0743(F), AZ0744(R), AZ0745(F), AZ0746(R), AZ0747(F), AZ0748(R) |
| Δ*ptsH* | pAL2318 | CTGTTTAAACTGCAGACTCT | DS159(F), DS160(R), DS161(F), DS162(R), DS163(F), DS164(R), DS165(F), DS166(R) |
| Δ*pgm* | pAL2038 | ATGGCAATCCACAATCGTGC | AZ0763(F), AZ0764(R), AZ0765(F), AZ0766(R), AZ0767(F), AZ0768(R) |

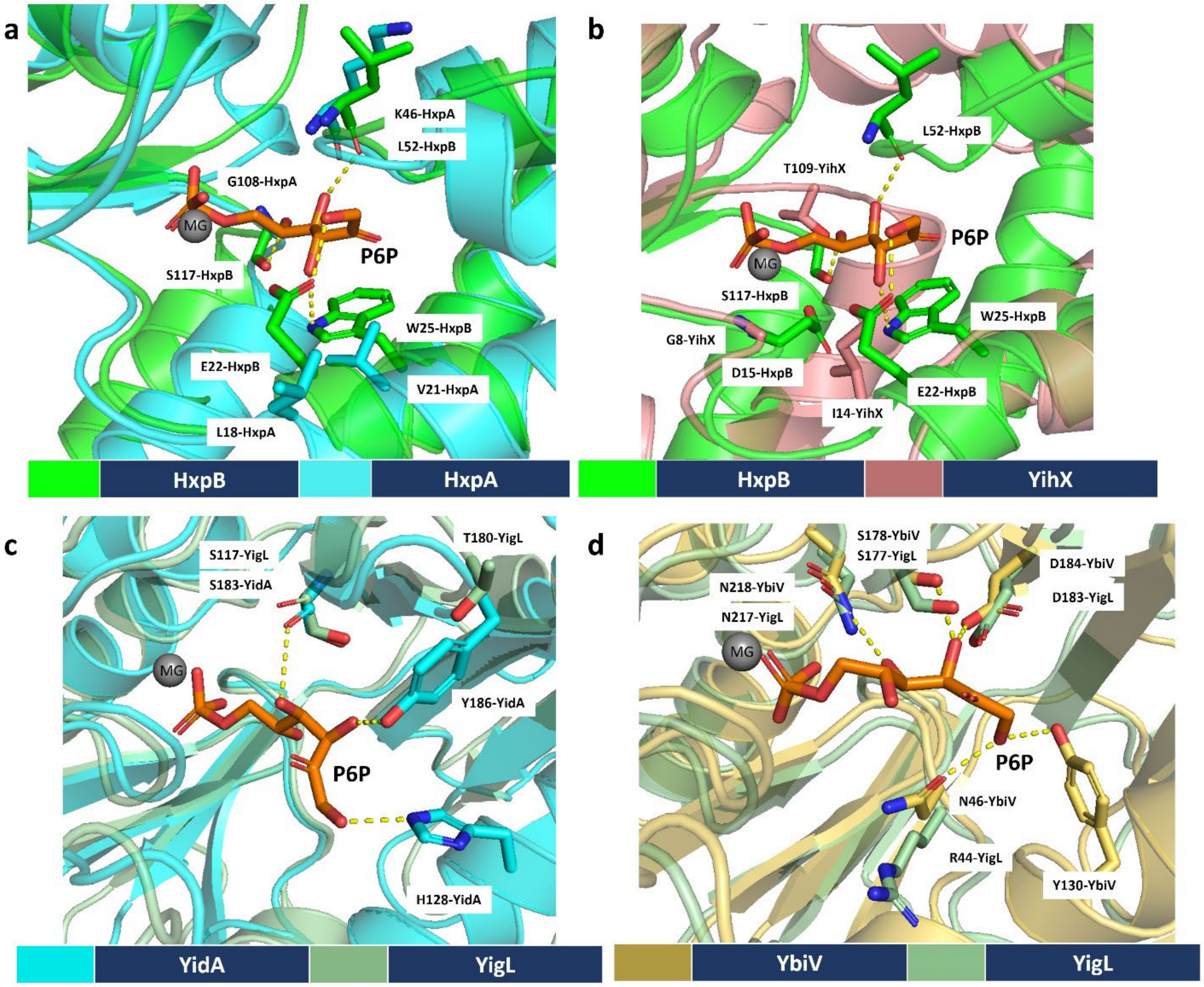


**Supplementary Figure 1. Predicted AlphaFold structures and P6P binding.**
**(a)** The structure of P6P-active HxpB (green, **Fig. 2b**) indicates that the residues E22, W25, L52, and S117 can hydrogen bond to P6P. The only hydrogen bond interaction for the structure of P6P-inactive HxpA (light blue, **Fig. 2b**) is the residue K46. No hydrogen bonds found between HxpA and the terminal hydroxyl group of P6P. **(b)** The only hydrogen bond interaction for P6P-inactive YihX (pink, **Fig. 2b**) is the residue T109. No hydrogen bonds found between YihX and terminal hydroxyl group of P6P. **(c)** The P6P-active structure of YidA (light blue, **Fig. 2b**), indicates that the residues H128, Y186, and S183 can hydrogen bond to P6P. The only hydrogen bond found in the structure of P6P-inactive YigL (light green, **Fig. 2b**) is residue S117. No hydrogen bonds found between YigL and terminal hydroxyl group of P6P. **(d)** The structure of P6P-active YbiV (gold, **Fig. 2b**) indicates that the residues N46, Y130, D184, S178, and N218 are hydrogen bonding to P6P. The only residues of P6P-inactive YigL (light green, **Fig. 2b**) hydrogen bonding with P6P are N217, S177, and D183. No hydrogen bonds found between YigL and terminal hydroxyl group of P6P.

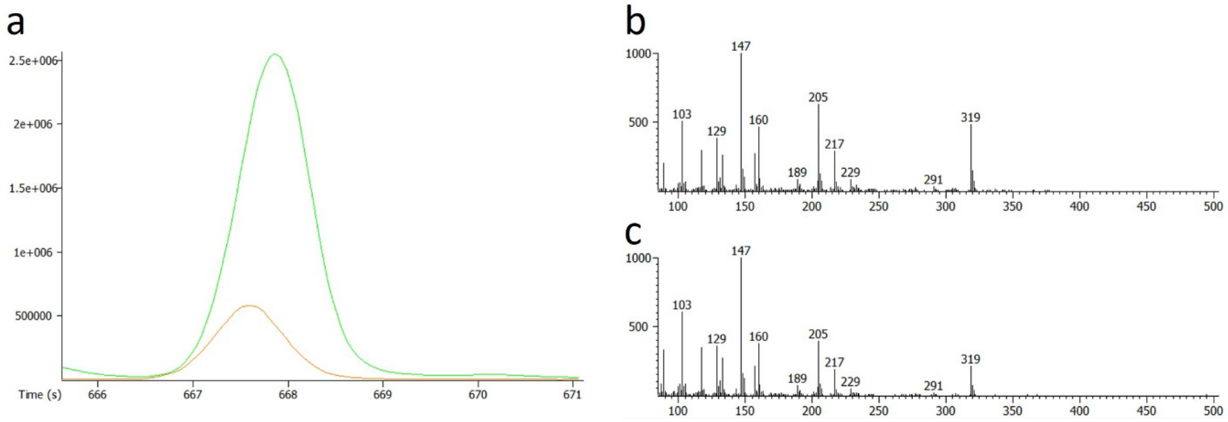


**Supplementary Figure 2. GC-MS identification of D-mannose side product.**
**(a)** The GC elution peak: the side product (green) and a mannose standard (brown). (**b&c**) The mass spectrum: the side product peak (**b**) and a mannose standard (**c**).

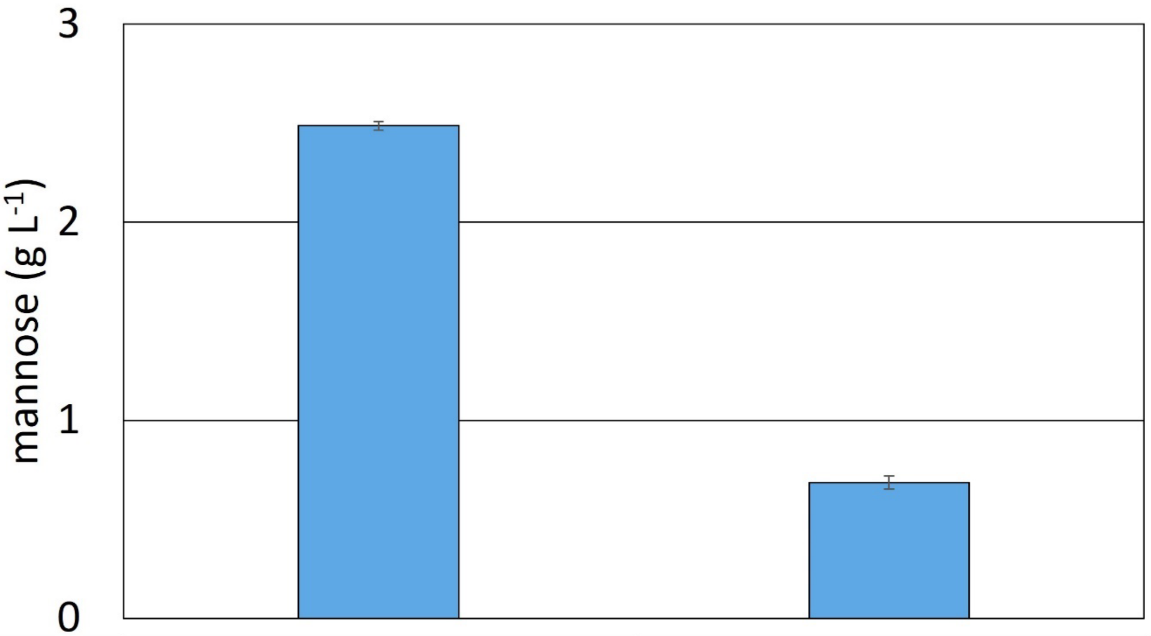


| strain | AL3756 (AL1050 Δ*zwf* Δ*pfkA* Δ*rpiB*) | AL3990 (AL1050 Δ*zwf* Δ*pfkA* Δ*rpiB* Δ*manA*) |
|---|---|---|
| $\Delta OD_{600}$ | 3.2 | 4.1 |

**Supplementary Figure 3. Effect of *manA* knockout on D-mannose production.**
To reduce mannose production, *manA* was deleted in AL3756 (**Supplementary Table 1**), generating AL3990 (**Supplementary Table 1**). Production plasmid pAL2001 (**Supplementary Table 2**) containing $P_{\mathrm{LlacO1}}$:*alsE-hxpB* was introduced into AL3756 and AL3990, generating Strain 1 and 2 (**Table 1**). Cultures were grown in M9P media supplemented with 10 g $L^{-1}$ glucose at 30 ˚C and induced with 1 mM IPTG. Error bars indicate s.d. (n = 3 biological replicates).

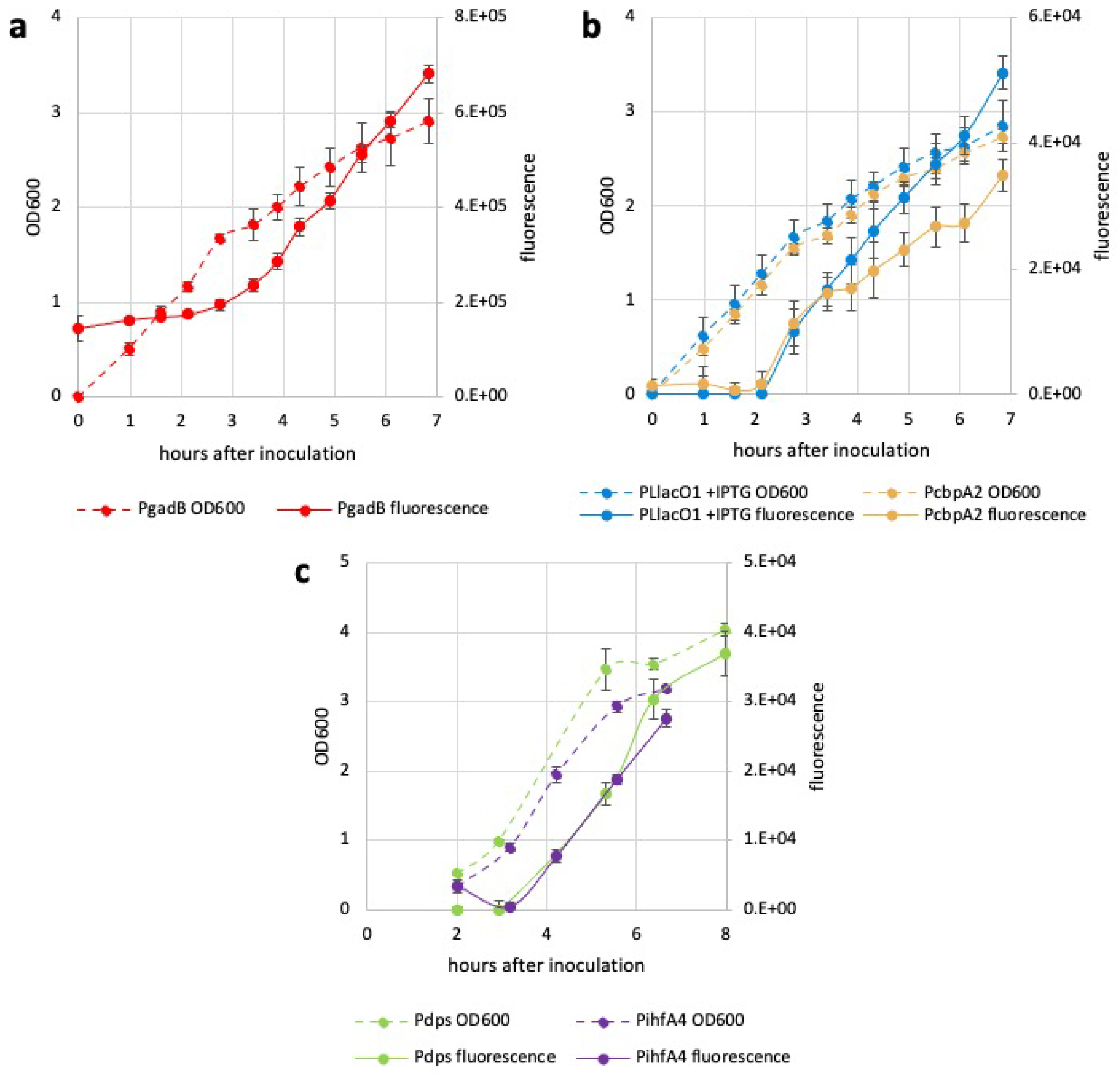


**Supplementary Figure 4. Characterization of the stationary phase promoters. a-c)** Fluorescence and $OD_{600}$ of strains with sfGFP expressed under promoters $P_{gadB}$ (**a**), $P_{cbpA2}$ (**b**), $P_{dps}$ (**c**), and $P_{ihfA4}$ (**c**) were monitored for timing and activity in comparison to $P_{LlacO1}$ induced with 1 mM IPTG. Error bars indicate s.d. (n = 3 biological replicates).

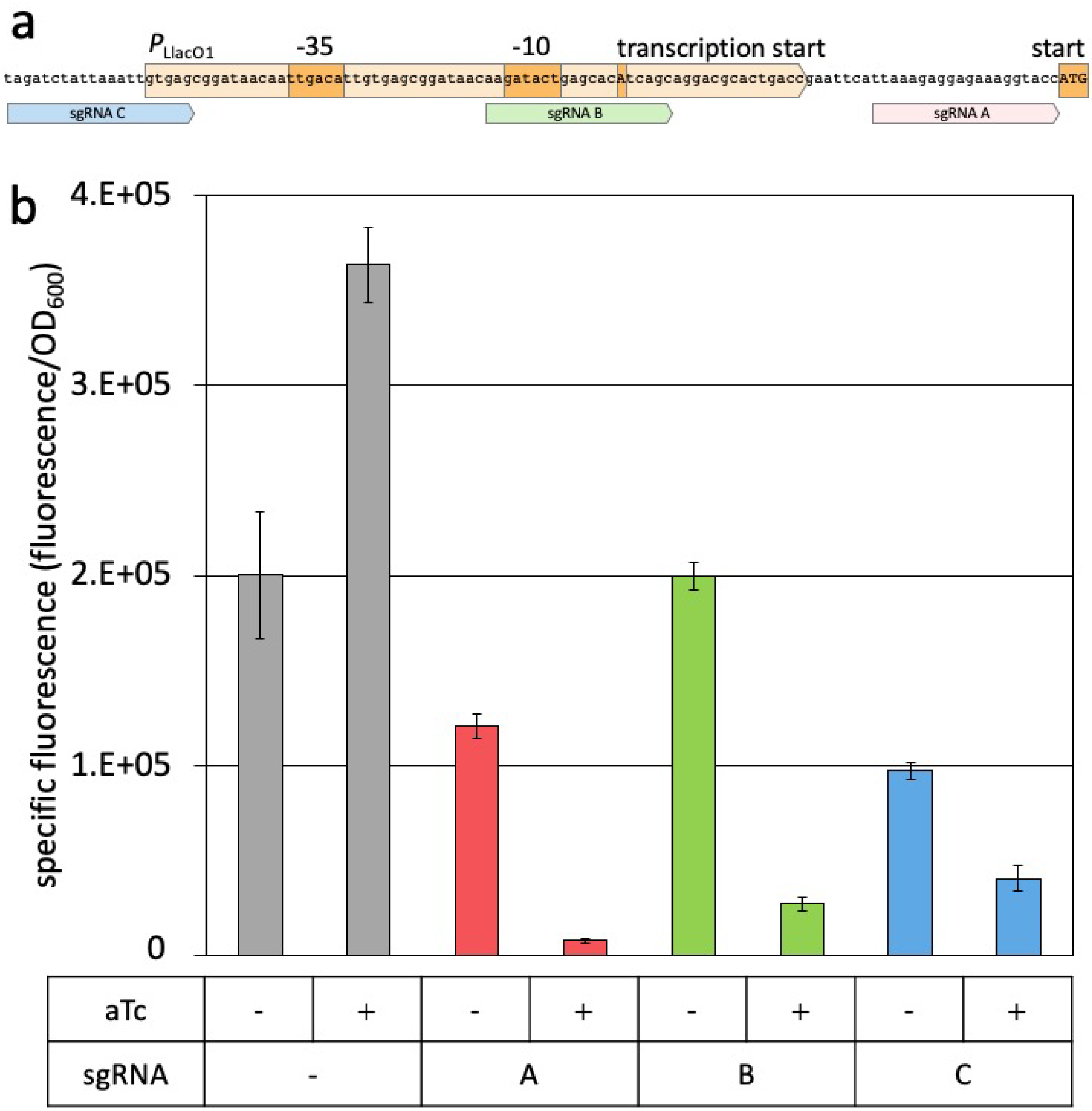


**Supplementary Figure 5. Inhibition of fluorescence by CRISPRi.**

**(a)** Three sgRNAs (A, B, and C) were designed for targeting $P_{\mathrm{LlacO1}}$:*sfgfp*. **(b)** The *dcas9* gene was cloned under the aTc-inducible promoter $P_{tet}$, generating plasmid pAL1952 (**Supplementary Table 2**). Constitutively expressed sgRNA A (pAL2066), B (pAL2173), and C (pAL2174) or sgRNA without targeting sequence (pAL2063) were individually cloned onto a plasmid containing $P_{\mathrm{LlacO1}}$:*sfgfp* (**Supplementary Table 2**). AL1050 with the CRISPRi system was cultured at 30 ˚C and fluorescence was measured at 0 and 4h after induction with 1 mM IPTG and with or without 100 ng $mL^{-1}$ aTc. Error bars indicate s.d. (n = 3 biological replicates).

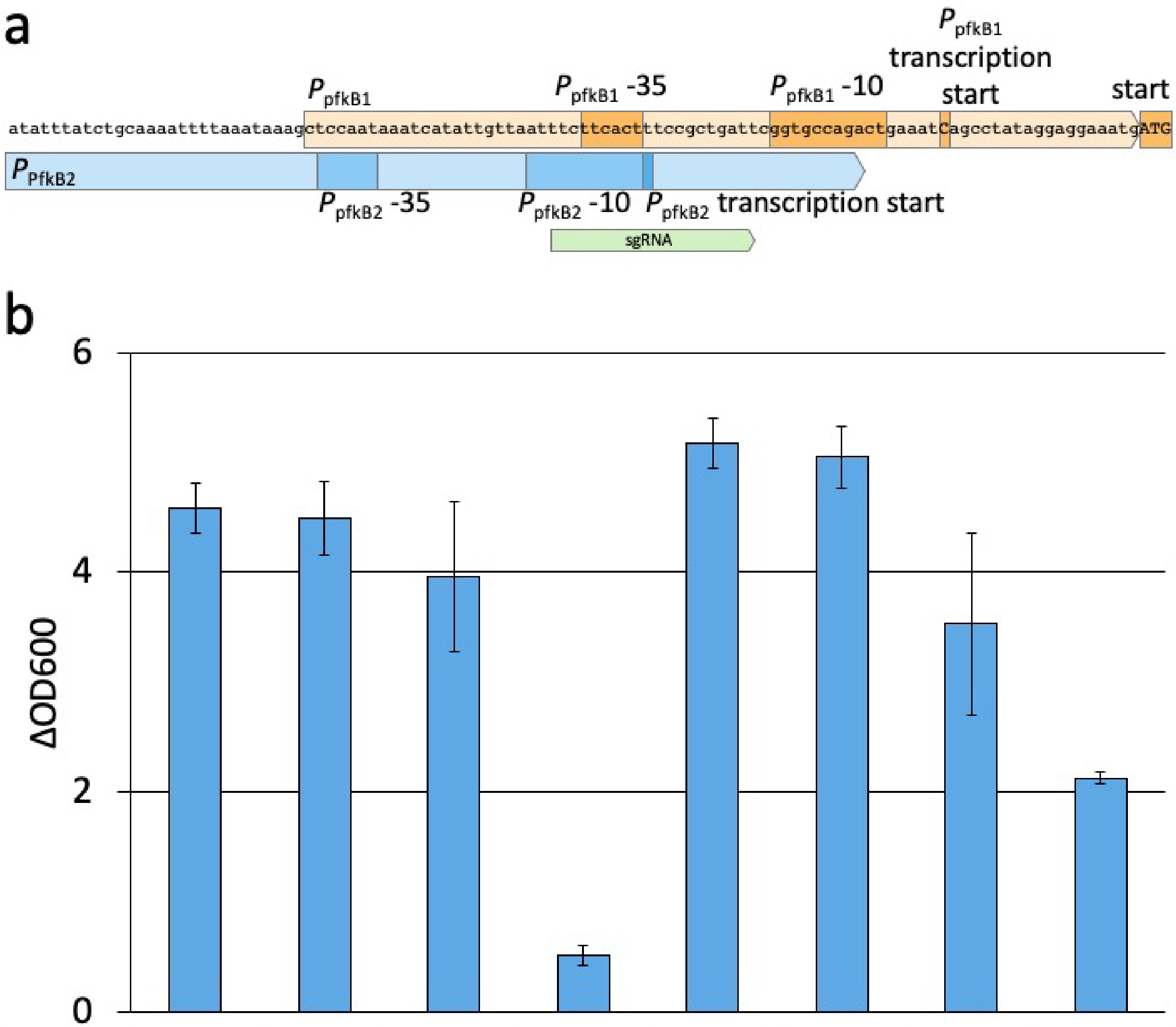


| aTc | - | + | - | + | - | + | - | + |
|---|---|---|---|---|---|---|---|---|
| Plasmid 1 | *P<sub>tet</sub>:dcas9* | | *P<sub>tet</sub>:dcas9* | | *P<sub>tet</sub>:dcas9, pTargetF-no target* | | *P<sub>tet</sub>:dcas9, pTargetF-pfkB* | |
| Plasmid 2 | *pTargetF-no target* | | *pTargetF-pfkB* | | - | | - | |

**Supplementary Figure 6. Growth inhibition with CRISPRi.**

The effect on growth of two different CRISPRi systems was tested in AL4186 (**Supplementary Table 1**). In the first CRISPRi system, *dcas9* and sgRNA are on a medium copy (p15A ori) and a high copy (ColE ori) plasmid, respectively. In the second CRISPRi system, both dcas9 and sgRNA are on the same plasmid (p15A ori). **(a)** sgRNA was designed to target the middle of the *pfkB* promoter region, $P_{pfkB1}$ and $P_{PfkB2}$. **(b)** Cells were grown at 30 ˚C and $OD_{600}$ was measured at 0 and 24 h. $\Delta OD_{600}$ indicates the difference in $OD_{600}$ at 0 h and 24 h. Error bars indicate s.d. (n = 3 biological replicates).

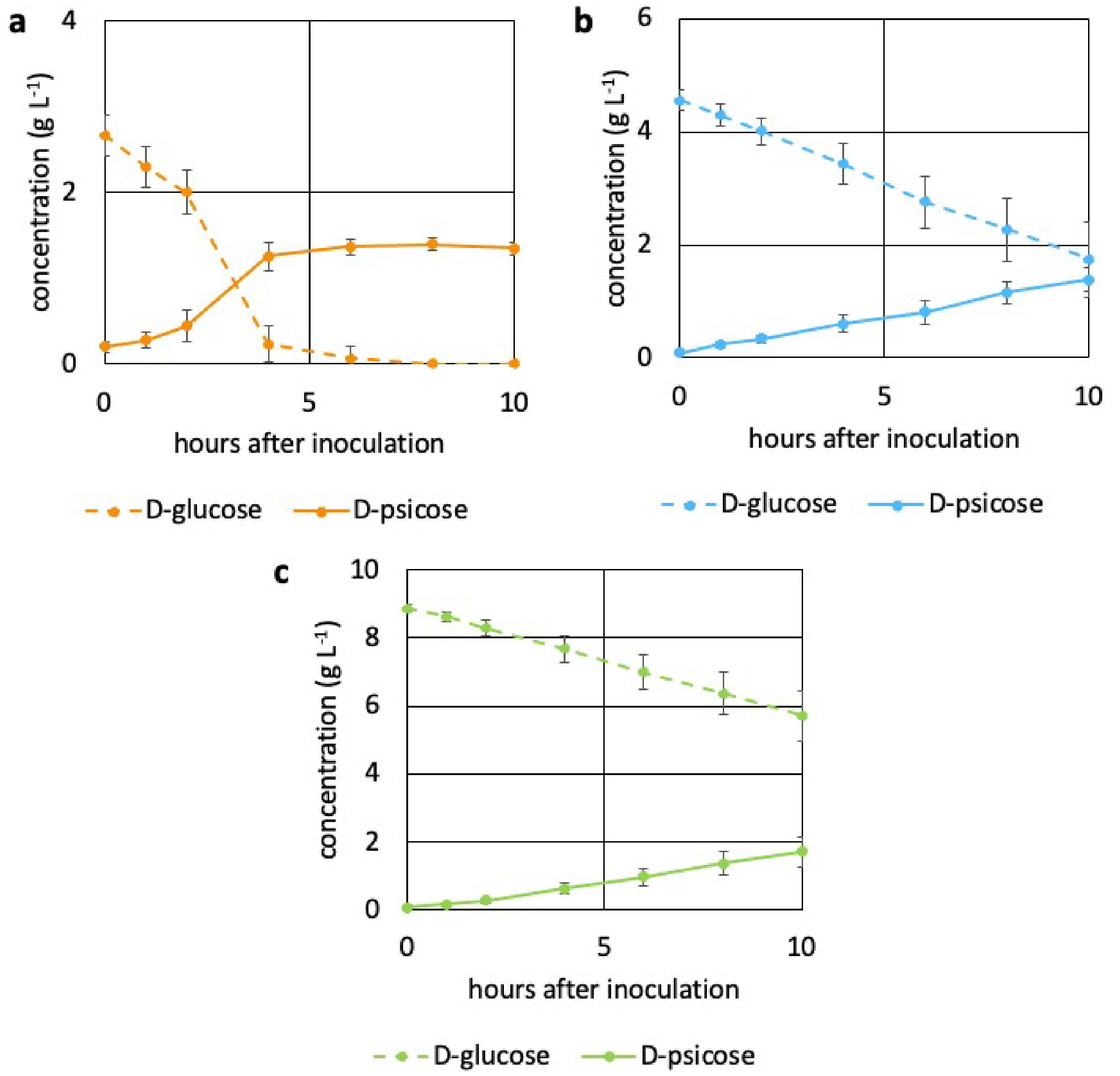


**Supplementary Figure 7. Glucose consumption and psicose production over time.**
D-glucose consumption and D-psicose production by Strain 7 (**Table 1**) was monitored in M9P media containing glucose at 3 **(a)**, 5 **(b)**, and 10 g $L^{-1}$ **(c)** at 30 ˚C for 10 h. Error bars indicate s.d. (n = 3 biological replicates).

**Rosetta XML:**

**All Rosetta simulations were run with Rosetta version 2018.24.post.dev+17.master.450949e**

<u>**Command**</u>

```
~/Rosetta/main/source/bin/rosetta_scripts.default.linuxgccrelease -database
~/Rosetta/main/database @flags -overwrite -parser:protocol docking.xml -out:path:all ./results
```

<u>**Example of the Rosetta script for docking(docking.xml)**</u>

```
<ROSETTASCRIPTS>
 <SCOREFXNS>
  <ScoreFunction name="myscore" weights="beta_genpot_cart.wts">
   <Reweight scoretype="coordinate_constraint" weight="1.0"/>
   <Reweight scoretype="atom_pair_constraint" weight="1.0"/>
   <Reweight scoretype="angle_constraint" weight="1.0"/>
   <Reweight scoretype="dihedral_constraint" weight="1.0"/>
   <Reweight scoretype="res_type_constraint" weight="1.0"/>
  </ScoreFunction>
  <ScoreFunction name="cstscore" weights="beta_genpot_cst.wts"/>
 </SCOREFXNS>
 <SCORINGGRIDS ligand_chain="X" width="20.0">
  <ClassicGrid grid_name="vdw" weight="1.0"/>
 </SCORINGGRIDS>
 <TASKOPERATIONS>
  <DetectProteinLigandInterface name="interface_detect" design="0" cut1="6.0" cut2="8.0"
cut3="10.0" cut4="12.0"/>
  <LimitAromaChi2 name="limchi2"/>
  <SetCatalyticResPackBehavior name="catres" fix_catalytic_aa="0"/>
 </TASKOPERATIONS>
 <FILTERS>
```

```
   <EnzScore name="allcst" score_type="cstE" scorefxn="cstscore" whole_pose="1"
energy_cutoff="2000"/>
  </FILTERS>
  <MOVERS>
   <AddOrRemoveMatchCsts name="cstadd" cst_instruction="add_new"/>
   <GALigandDock name="GAdock" runmode="refine" scorefxn="myscore" padding="6.0"
sidechains="aniso" final_exact_minimize="bbsc2" rotprob="0.9" rotEcut="100">
     <Stage repeats="100" npool="100"/>
   </GALigandDock>
  </MOVERS>
<PROTOCOLS>
   <Add mover_name="cstadd"/>
   <Add mover="GAdock"/>
   <Add filter="allcst"/>
  </PROTOCOLS>
</ROSETTASCRIPTS>
```

**Flags:**

```
-beta_cart
-run:preserve_header
-run:version
-nblist_autoupdate
-linmem_ig 10
-jd2::enzdes_out
-chemical:exclude_patches LowerDNA  UpperDNA Cterm_amidation VirtualBB ShoveBB VirtualDNAPhosphate VirtualNTerm CTermConnect sc_orbitals pro_hydroxylated_case1 pro_hydroxylated_case2 ser_phosphorylated thr_phosphorylated  tyr_phosphorylated tyr_sulfated lys_dimethylated lys_monomethylated  lys_trimethylated lys_acetylated
```

glu_carboxylated cys_acetylated tyr_diiodinated N_acetylated C_methylamidated MethylatedProteinCterm

-enzdes::minimize_all_ligand_torsions 5.0

-enzdes::detect_design_interface

-packing::extrachi_cutoff 1

-packing::ex1

-packing::ex2

-packing::ex1aro:level 6

-packing::use_input_sc

-packing::flip_HNQ

-packing::no_optH false

-packing::optH_MCA false

-enzdes::favor_native_res 2

-enzdes::bb_min_allowed_dev 0.05

-extra_res_fa 114.params

**Catalytic Constraints**

#Aspartic acid with Phosphate on P6P

CST::BEGIN

TEMPLATE:: ATOM_MAP: 1 atom_name: P O2 C3

TEMPLATE:: ATOM_MAP: 1 residue3: 114

TEMPLATE:: ATOM_MAP: 2 atom_name: OD1 CG OD2

TEMPLATE:: ATOM_MAP: 2 residue3: ASP

CONSTRAINT:: distanceAB: 1.7 0.4 100 1

CST::END

#Lysine with Oxygen on P6P

CST::BEGIN

TEMPLATE:: ATOM_MAP: 1 atom_name: O6 P O2

TEMPLATE:: ATOM_MAP: 1 residue3: 114

```
  TEMPLATE::   ATOM_MAP: 2 atom_name: NZ CE CD
  TEMPLATE::   ATOM_MAP: 2 residue3: LYS
  CONSTRAINT:: distanceAB:    2.7   0.4  100  0
  CONSTRAINT::    angle_A:   109.5   10.0   50  360.
CST::END
#Catalytic Asp with P6P
CST::BEGIN
  TEMPLATE::   ATOM_MAP: 1 atom_name: O2 C3 C1
  TEMPLATE::   ATOM_MAP: 1 residue3: 114
  TEMPLATE::   ATOM_MAP: 2 atom_name: OD2 CG CB
  TEMPLATE::   ATOM_MAP: 2 residue3: ASP
  CONSTRAINT:: distanceAB:    3.0   0.4  100  0
  CONSTRAINT::    angle_A:   109.5   10.0   50  360.
  CONSTRAINT::    angle_B:   120.0   10.0   50  360.
  CONSTRAINT::  torsion_B:   0.0   10.0   50  180.
CST::END
#Aspartic Acid with MG
CST::BEGIN
  TEMPLATE::   ATOM_MAP: 1 atom_name: MG V1 V2
  TEMPLATE::   ATOM_MAP: 1 residue3: MG
  TEMPLATE::   ATOM_MAP: 2 atom_name: OD1 CG OD2
  TEMPLATE::   ATOM_MAP: 2 residue3: ASP
  CONSTRAINT:: distanceAB:    2.1   0.3  100  0
CST::END
```

**Ligand Params**

```
NAME 114
IO_STRING 114 Z
TYPE LIGAND
```

AA UNK

ATOM P    PG3  X     0.754

ATOM O    Ohx  X    -0.709

ATOM O1   Ohx  X    -0.606

ATOM O2   OG3  X    -0.512

ATOM O3   Ohx  X    -0.618

ATOM O4   Oal  X    -0.639

ATOM O5   Ohx  X    -0.601

ATOM O6   OG2  X    -0.695

ATOM O7   OG2  X    -0.695

ATOM O8   OG2  X    -0.695

ATOM C    CSp  X     0.194

ATOM C1   CSp  X     0.072

ATOM C2   CSp  X     0.036

ATOM C3   CSp  X     0.341

ATOM C4   CDp  X     0.617

ATOM C5   CSp  X    -0.076

ATOM H    HO   X     0.510

ATOM H1   HO   X     0.353

ATOM H2   HO   X     0.400

ATOM H3   HO   X     0.394

ATOM H4   HC   X    -0.007

ATOM H5   HC   X     0.028

ATOM H6   HC   X     0.011

ATOM H7   HC   X    -0.049

ATOM H8   HC   X    -0.049

ATOM H9   HC   X     0.120

ATOM H10  HC   X     0.120

BOND_TYPE P O2 1

BOND_TYPE P O6 1

BOND_TYPE P O7 1

BOND_TYPE P O8 1

BOND_TYPE O C 1

BOND_TYPE O1 C1 1

BOND_TYPE O2 C3 1

BOND_TYPE O3 C2 1

BOND_TYPE O5 C5 1

BOND_TYPE C C1 1

BOND_TYPE C C2 1

BOND_TYPE C1 C3 1

BOND_TYPE C2 C4 1

BOND_TYPE C4 C5 1

BOND_TYPE O H 1

BOND_TYPE O1 H1 1

BOND_TYPE O3 H2 1

BOND_TYPE O5 H3 1

BOND_TYPE C H4 1

BOND_TYPE C1 H5 1

BOND_TYPE C2 H6 1

BOND_TYPE C3 H7 1

BOND_TYPE C3 H8 1

BOND_TYPE C5 H9 1

BOND_TYPE C5 H10 1

BOND_TYPE O4 C4 2

NBR_ATOM C1

NBR_RADIUS 13.06307

```
ICOOR_INTERNAL  C1      0.000000    0.000000    0.000000 C1   C    O
ICOOR_INTERNAL  C       0.000000  180.000000    1.539826 C1   C    O
ICOOR_INTERNAL  O       0.000000   70.068245    1.424293 C    C1   O
ICOOR_INTERNAL  O1    175.072457   70.035011    1.426376 C1   C    O
ICOOR_INTERNAL  C2    125.793681   64.520295    1.536374 C    C1   O
ICOOR_INTERNAL  C3     55.126979   68.163861    1.528670 C1   C    O
ICOOR_INTERNAL  C4    -61.215375   64.440474    1.527953 C2   C    C1
ICOOR_INTERNAL  C5     93.089362   62.929796    1.516494 C4   C2   C
ICOOR_INTERNAL  O4    -85.481488   58.416615    1.231850 C4   C2   C
ICOOR_INTERNAL  O2   -178.592766   71.206513    1.424434 C3   C1   C
ICOOR_INTERNAL  O3    177.536152   71.936295    1.421694 C2   C    C1
ICOOR_INTERNAL  O5    179.754769   69.551723    1.413694 C5   C4   C2
ICOOR_INTERNAL  P     179.986649   61.751098    1.615936 O2   C3   C1
ICOOR_INTERNAL  O6     53.061783   76.178219    1.617445 P    O2   C3
ICOOR_INTERNAL  O7    -54.296189   77.286748    1.616927 P    O2   C3
ICOOR_INTERNAL  O8    179.985033   64.889432    1.500698 P    O2   C3
ICOOR_INTERNAL  H     -51.420370   70.564959    0.970676 O    C    C1
ICOOR_INTERNAL  H1     57.754946   70.476868    0.970492 O1   C1   C
ICOOR_INTERNAL  H2    -54.213687   70.536654    0.969609 O3   C2   C
ICOOR_INTERNAL  H3    179.998800   70.536111    0.969576 O5   C5   C4
ICOOR_INTERNAL  H4   -119.392551   74.742001    1.070082 C    C1   O
ICOOR_INTERNAL  H5    -64.246196   72.940474    1.070915 C1   C    O
ICOOR_INTERNAL  H6     55.455278   74.144376    1.069844 C2   C    C1
ICOOR_INTERNAL  H7    -58.670477   70.337530    1.070416 C3   C1   C
ICOOR_INTERNAL  H8     61.107923   70.114283    1.070185 C3   C1   C
ICOOR_INTERNAL  H9    -60.118012   70.793540    1.069810 C5   C4   C2
ICOOR_INTERNAL  H10    60.140744   71.055280    1.069784 C5   C4   C2
CHI   1   C3   O2   P   O6
```

CHI 2 C2 C4 C5 O5

CHI 3 C1 C3 O2 P

CHI 4 O1 C1 C O

CHI 5 O C C2 O3

**Supplementary references**


1. Baba, T. *et al.* Construction of *Escherichia coli* K-12 in-frame, single-gene knockout mutants: The Keio collection. *Mol. Syst. Biol.* **2**, 0008 (2006).
2. Yoneda, H., Tantillo, D. J. & Atsumi, S. Biological Production of 2-Butanone in *Escherichia coli*. *ChemSusChem* **7**, 92–95 (2014).
3. Zhang, A. *et al.* Microbial production of human milk oligosaccharide lactodifucotetraose. *Metab. Eng.* **66**, 12–20 (2021).
